# Asparagine restriction enhances CD8^+^ T cell metabolic fitness and antitumoral functionality through an NRF2-dependent stress response

**DOI:** 10.1038/s42255-023-00856-1

**Published:** 2023-08-07

**Authors:** J. N. Rashida Gnanaprakasam, Bhavana Kushwaha, Lingling Liu, Xuyong Chen, Siwen Kang, Tingting Wang, Teresa A. Cassel, Christopher M. Adams, Richard M. Higashi, David A. Scott, Gang Xin, Zihai Li, Jun Yang, Andrew N. Lane, Teresa W.-M. Fan, Ji Zhang, Ruoning Wang

**Affiliations:** 1 Center for Childhood Cancer, Hematology/Oncology & BMT, Abigail Wexner Research Institute at Nationwide Children’s Hospital, Department of Pediatrics at The Ohio State University, Columbus, OH USA; 2 Center for Environmental and Systems Biochemistry, Department of Toxicology and Cancer Biology, Markey Cancer Center, University of Kentucky, Lexington, KY USA; 3 Division of Endocrinology, Diabetes, Metabolism and Nutrition, Mayo Clinic, Rochester, MN USA; 4 Cancer Metabolism Core, Sanford Burnham Prebys Medical Discovery Institute, La Jolla, CA USA; 5 Department of Microbial Infection and Immunity, Pelotonia Institute for Immuno-Oncology, The Ohio State University, Columbus, OH USA; 6 Department of Surgery, St. Jude Children’s Research Hospital, Memphis, TN USA; 7 Department of Pathology, College of Medicine, The University of Tennessee Health Science Center, Memphis, TN USA; 8 Herman B Wells Center for Pediatric Research, Indiana University School of Medicine, Indianapolis, IN USA

**Keywords:** Immunology, Immunotherapy, Metabolism

## Abstract

Robust and effective T cell immune surveillance and cancer immunotherapy require proper allocation of metabolic resources to sustain energetically costly processes, including growth and cytokine production. Here, we show that asparagine (Asn) restriction on CD8^+^ T cells exerted opposing effects during activation (early phase) and differentiation (late phase) following T cell activation. Asn restriction suppressed activation and cell cycle entry in the early phase while rapidly engaging the nuclear factor erythroid 2-related factor 2 (NRF2)-dependent stress response, conferring robust proliferation and effector function on CD8^+^ T cells during differentiation. Mechanistically, NRF2 activation in CD8^+^ T cells conferred by Asn restriction rewired the metabolic program by reducing the overall glucose and glutamine consumption but increasing intracellular nucleotides to promote proliferation. Accordingly, Asn restriction or NRF2 activation potentiated the T cell-mediated antitumoral response in preclinical animal models, suggesting that Asn restriction is a promising and clinically relevant strategy to enhance cancer immunotherapy. Our study revealed Asn as a critical metabolic node in directing the stress signaling to shape T cell metabolic fitness and effector functions.

## Abstract (web summary)

Gnanaprakasam et al. study the amino acid requirements during different phases of T cell activation and show how asparagine restriction imparts opposing effects during early and late phases of T cell maturation through an NRF2-dependent mechanism.

## Main

The exquisite specificity, amplitude and quality of the T cell response are critical for immune surveillance and cancer immunotherapy. However, the T cell response is a metabolically costly process. It is often constrained by the metabolic landscape of the tissue microenvironment and the rapidly proliferating pathogens or cancer cells that compete for nutrient resources with the host. T cell activation rapidly engages the central carbon catabolic pathways, including glycolysis, the pentose phosphate pathway (PPP) and the tricarboxylic acid (TCA) cycle, to prepare cells for growth, differentiation and immune defense^1–4^. An optimal allocation of limited energy and nutrient resources among growth, repair and production of effector molecules is required to maximize T cell-mediated responses. The largest constituent of cell mass comes from amino acids in proteins. Amino acids are the building blocks of protein, sources of carbon or nitrogen for energy, anabolic substrates, and signaling molecules. T cells rely solely on exogenous sources of essential amino acids to grow. Although T cells can synthesize all of the non-essential amino acids (NEAAs), robust proliferation and effector response requires an exogenous supply of NEAAs^5,6^. The bioavailability of amino acids is tightly coupled with the central carbon metabolism and cell signaling transduction that shape T cell proliferation, differentiation and immunological functions^1^. CD8^+^ effector T (T_eff_) cells play a major role in antitumor immunity and elicit antitumor activity by directly recognizing and killing antigen-presenting tumor cells and orchestrating numerous adaptive and innate immune responses. However, tumors can co-opt various immunosuppressive mechanisms that act in concert to foster an immune-tolerant microenvironment and thus escape the T cell-mediated antitumor immune response^7,8^. As activated T cells share metabolic characteristics with tumor cells^7,8^, the metabolically demanding cancer cells restrict the function of T_eff_ cells by competing for nutrients and producing immunosuppressive metabolites^7^. The development of CD8^+^ T_eff_ responses can be broadly classified into two distinct and sequential phases: an activation phase (early) in which CD8^+^ T cells accumulate cellular mass and prepare to divide, followed by a differentiation phase (late) in which CD8^+^ T cells rapidly expand and differentiate into effector cells^9^. Shifts in energy and carbon expenditure accompany the early-to-late phase transition to support different biological activities and effector functions. A better understanding of the amino acid dependence of CD8^+^ T cells and the metabolic interplay within the tumor microenvironment will enable us to devise rational and effective approaches to improve T cell metabolic fitness and cancer immunotherapy.

## Results

### Profiling the dependency of CD8^+^ T cells on extracellular amino acids

**Fig. 1:**
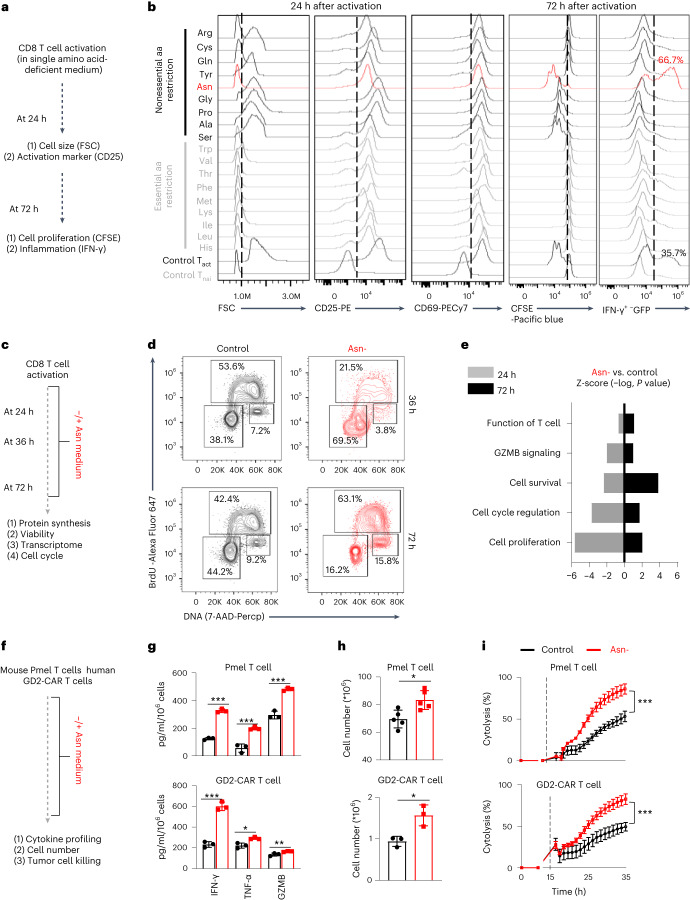
Asn restriction enhances CD8^+^ T cell proliferation and effector functions in vitro. **a**, Schematic diagram of comprehensively profiled T cell activation, proliferation and IFN-γ production under single amino acid-deficient conditions. FSC, forward scatter. **b**, CD8^+^ T cells were activated in a complete medium or indicated single amino acid-deficient medium. Cell size (FSC) and cell surface expression of CD25 and CD69 were determined by flow cytometry in the early phase (24 h). Proliferation (CFSE) and intercellular expression of IFN-γ were determined by flow cytometry in the late phase (72 h). Flow plots are representative of two independent experiments. aa, amino acid; T_nai_, Naive CD8^+^ T cells; T_act_, Active CD8^+^ T cells. **c**, Schematic diagram of sample collection and assays. **d**,**e**, CD8^+^ T cells were activated in a medium with or without Asn and were collected in the early phase (24–36 h) and late phase (72 h). The cell cycle profile (**d**) was determined by BrdU incorporation and 7-AAD staining by flow cytometry. The numbers indicate the percentage of cells in the cell cycle stage (*n* = 3 experimental replicates). Data are representative of three independent experiments. Comparative differently expressed gene signatures (**e**) were determined by the Ingenuity pathway analysis (IPA) of RNA-seq and listed according to their z-score. Data are representative of one experiment. **f**, Schematic diagram of in vitro assays. **g**–**i**, Indicated proteins in culture supernatants were quantified by ELISA and LEGENDplex (**g**) (*n* = 3 experimental replicates; upper panel: *P* < 0.0001, *P* = 0.015 and *P* = 0.0004 for IFN-γ, TNF-α and GZMB, respectively; lower panel: *P* = 0.002, *P* = 0.0111 and *P* = 0.0274 for IFN-γ, TNF-α and GZMB, respectively). The cell number was determined by a cell counter (**h**) (*n* = 3 experimental replicates for GD2-CAR T cells and *n* = 5 experimental replicates for Pmel T cells; *P* = 0.0108 and *P* = 0.0176 for the upper and lower panels, respectively). The cytolysis was determined by eSight (**i**) (*n* = 3 experimental replicates; *P* = < 0.0001). Data in **g**–**i** are representative of three independent experiments. Error bars are mean ± s.d. *P < 0.05; **P < 0.01; ***P < 0.001. Statistical differences were determined by paired two-tailed Student’s *t-*test (**e**), unpaired two-tailed Student’s *t-*test (**g**, **h**) and two-way ANOVA (**i**). Source data

To systematically assess the amino acid dependence of T cells, we prepared single amino acid-deficient media by removing each of the 18 amino acids from the culture media. Glutamate (Glu) or aspartate (Asp) restriction was excluded from the experiment because non-neuronal cell types, including T cells, cannot transport these two amino acids^10^. Then, we activated CD8^+^ T cells under single amino acid-deficient conditions and profiled cell activation marker and size in the initial activation phase (24 h) and proliferation and interferon-γ (IFN-γ) production in the differentiation phase (72 h) (Fig. 1a). The activation markers CD25 and CD69 were induced after activation under every single amino acid restriction condition, albeit to various degrees (Fig. 1b). However, cell growth in the early phase, proliferation, and cytokine production in the late phase strictly require all essential amino acids and several NEAAs, including glutamine (Gln), arginine (Arg), cysteine (Cys) and tyrosine (Tyr) (Fig. 1b). These findings align with the known role of NEAAs in supporting the bioenergetic and biosynthetic activities of T cells^1,5,6,11–14^. In line with recent findings that extracellular Asn is required for T cell activation^15,16^, Asn restriction reduced cell size and the level of activation markers (Fig. 1b). In contrast, Asn restriction enhanced proliferation and the production of IFN-γ^+^ CD8^+^ T_eff_ cells (Fig. 1b), indicating that Asn restriction elicited opposing effects on CD8^+^ T cells during activation and differentiation.

### Asn restriction enhances proliferation and effector function

**Extended Data Fig. 1:**
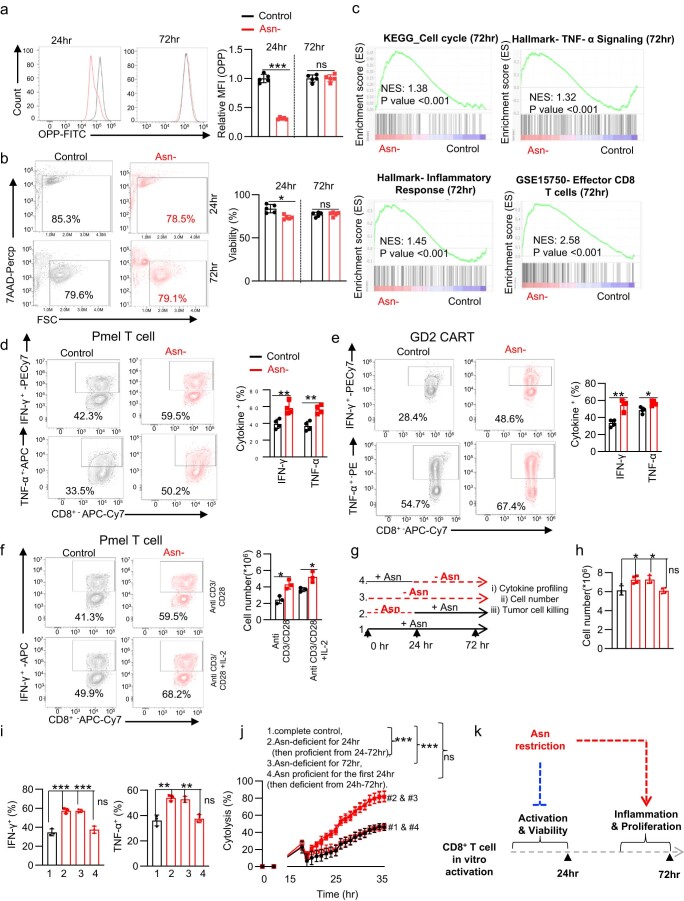
Asn restriction suppresses CD8^+^ T cell activation but enhances inflammation and proliferation. **a-b**) CD8^+^ T cells were activated in a medium with or without Asn and were collected in the early phase (24 hr) and late phase (72 hr). The protein content (**a**) (n = 3 experimental replicates, P < 0.0001 and P = 0.9368 at 24 and 72 h respectively) and cell viability (**b**) (n = 5 experimental replicates, (P = 0.003 and P = 0.7328 at 24 and 72 h respectively) was determined by 7AAD staining by flow cytometry. Data are representative of 3 independent experiments. **c**) Cell cycle, TNF-α, inflammatory response, and effector T cell function associated gene sets were analyzed by GSEA. **d-f**) Indicated cytokine was quantified by intracellular staining by flow cytometry as indicated condition (n = 4 experimental replicates, Pmel T cells: P = 0.007 and P = 0.002 for IFN-γ and TNF-α respectively; GD2 CAR-T: P = 0.0013 and P = 0.01 for IFN-γ and TNF-α respectively; Pmel T cells: P = 0.01 and P = 0.19 for no IL-2 and IL-2 respectively). **g**) Schematic diagram of the experiment. **h**) cell number (n = 4 experimental replicates, P = 0.01, P = 0.01 and P = 0.9 for group 1 vs. 2, 1 vs. 3 and 1 vs. 4 respectively). **i**) The indicated cytokines were quantified by flow cytometry (n = 3 experimental replicates, IFN-γ: P = 0.0009, P = 0.0006, and P = 0.3992; TNF-α: P = 0.0032, P = 0.004, and P = 0.668 for group 1 vs. 2, 1 vs. 3 and 1 vs. 4 respectively). **j**) The cytolysis was determined by eSight (n = 3 experimental replicates, P < 0.0001, P < 0.0001 and P = 0.675 in 1 vs. 2, 1 vs. 3 and 1 vs. 4 respectively). Data from **d-j** are representative of 3 independent experiments. Error bars represent mean ± SD. *P < 0.05, **P < 0.01, *** P < 0.001, Statistical differences were determined by unpaired Two-tail Student’s t-test (a, b, d-f and h-i) and Two-way ANOVA (**j**). **k**) Conceptual model showing that Asn restriction suppressed T cell activation but conferred robust proliferation and effector function to CD8^+^ Teff cells at the late time point. Source data

Next, we comprehensively assessed the effects of Asn restriction on CD8^+^ T cell activation and differentiation (Fig. 1b). Asn restriction significantly delayed cell cycle progression from G0/1 to S phase (Fig. 1d) and reduced protein content (Extended Data Fig. 1a), which was associated with moderately reduced cell viability (Extended Data Fig. 1b) at the early time point (24 h) (Fig. 1c). In contrast, Asn restriction increased the percentage of cells in the S phase (Fig. 1d) and increased total cell numbers without affecting the level of protein content and cell viability at the late time point (72 h) (Extended Data Fig. 1a, b). To gain mechanistic insights into the effects of Asn restriction on CD8^+^ T cells, we performed RNA sequencing (RNA-seq) in cells collected at the early and late time points, which revealed opposing gene enrichment patterns for the genes involved in the cell cycle/proliferation, cell survival and T cell function/signaling at the early time point versus the late time point (Fig. 1e). Notably, cell cycle-promoting genes, inflammatory signaling genes and CD8^+^ effector genes are significantly enriched under Asn restriction compared with the control condition in the late time point (Extended Data Fig. 1c), prompting our closer examination of the effect of Asn restriction in CD8^+^ T_eff_ cell-mediated antitumoral response. We generated antigen-specific mouse T_eff_ cells using a major histocompatibility complex (MHC) class I-restricted premelanosome protein (Pmel)-1 T cell receptor model (Fig. 1f)^17^. We also generated disialoganglioside (GD2)-specific chimeric antigen receptor (CAR) human T_eff_ cells (Fig. 1f)^18^. Pmel T_eff_ cells recognized Pmel-17 (mouse homolog of human SIVL/gp100) in mouse melanoma, whereas GD2-CAR T cells recognized GD2 expressed in tumors with neuroectodermal origin. Asn restriction increased the expression and production of proinflammatory cytokines (tumor necrosis factor-α (TNF-α) and IFN-γ) and effector molecule (granzyme B (GZMB)) in Pmel T_eff_ cells and GD2-CAR T cells (Fig. 1g and Extended Data Fig. 1d, e). In addition, Asn restriction increased the T cell number in these two models (Fig. 1h). We then co-cultured Pmel T_eff_ cells with Pmel^+^ B16F10-gp100 mouse melanoma cells, and GD2-CAR T cells with GD2^+^ LAN-1 human neuroblastoma cells. Asn restriction significantly enhanced the tumor-killing activities of T cells in both models (Fig. 1i). Next, we asked if other factors could change the effects of Asn restriction on T cells. Exogenous interleukin-2 (IL-2) is dispensable for Asn restriction-induced IFN-γ production increase or cell number increase (Extended Data Fig. 1f). Moreover, Asn restriction during the first 24-h period upon activation is sufficient to increase cell number, cytokine production and cytolysis activity of T cells collected at the 72-h time point (Extended Data Fig. 1g–j). Our data suggested that Asn restriction at the early stage suppressed T cell activation but conferred robust proliferation and effector function to CD8^+^ T_eff_ cells at the late stage (Extended Data Fig. 1k).

### Asn restriction induces Asn biosynthesis in CD8^+^ T cells

**Fig. 2:**
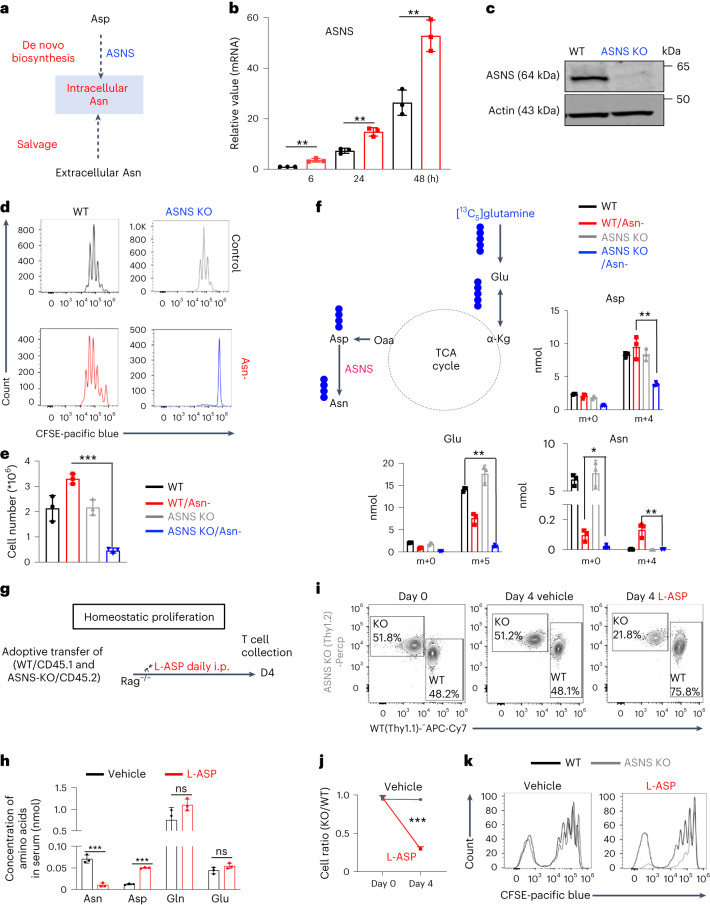
Asn restriction renders CD8^+^ T cells dependent on Asn de novo synthesis. **a**, Conceptual diagram of maintaining the intracellular Asn pool in T cells. **b**, ASNS mRNA expression in the indicated groups was determined by quantitative PCR (qPCR) (*n* = 3 experimental replicates; *P* = 0.0036, *P* = 0.0027 and *P* = 0046 for 6 h, 24 h and 48 h, respectively). Data are representative of two independent experiments. **c**, The indicated protein was determined by immunoblot. Data are representative of three independent western blot experiments. **d**,**e**, CD8^+^ T cells with the indicated genotype were activated in a medium with or without Asn. Cell proliferation (CFSE) (**d**) was determined by flow cytometry. Cell number (**e**) was determined by a cell counter (*n* = 3 experimental replicates; *P* < 0.0001). Data in **d** and **e** are representative of three independent experiments. **f**, Diagram of [^13^C_5_]glutamine catabolism through entering the downstream TCA cycle and Asn biosynthesis. Filled circles denote the ^13^C label of all carbons of indicated metabolites derived from [^13^C_5_]glutamine catabolism (left panel). Metabolites in the indicated groups were analyzed by GC–MS (right panel); numbers on the *x* axis represent those of ^13^C atoms in given metabolites, and numbers on the *y* axis represent the levels of the metabolites (nmol per protein). *n* = 3 experimental replicates; *P* = 0.0048, *P* = 0.0012 and *P* = 0.0086 for Asp, Glu and Asn, respectively. Data are representative of one experiment. **g**, Schematic diagram of in vivo competitive proliferation. **h**, Mouse serum Asn, Asp, Gln and Glu levels were quantified by GC–MS (*n* = 3 mice per group; *P* = 0.0006, *P* < 0.0001, *P* = 0.134 and *P* = 0.8018 for Asn, Asp, Gln and Glu, respectively). Data are representative of two independent experiments. **i**–**k**, The donor cell ratios before and after adoptive transfer were determined by surface staining of isogenic markers (**i**), and cell ratio was calculated (**j**) (*P* = 0.0005). Cell proliferation was determined by CFSE dilution (**k**). Data in **i**–**k** are representative of three independent experiments (*n* = 3 mice per group). Error bars represent mean ± s.d. *P < 0.05; **P < 0.01; ***P < 0.001; ns, not significant. Statistical differences were determined by unpaired two-tailed Student’s *t-*test (**b**, **e**, **f** and **h**) and paired two-tailed Student’s *t-*test (**j**). Source data

**Extended Data Fig. 2:**
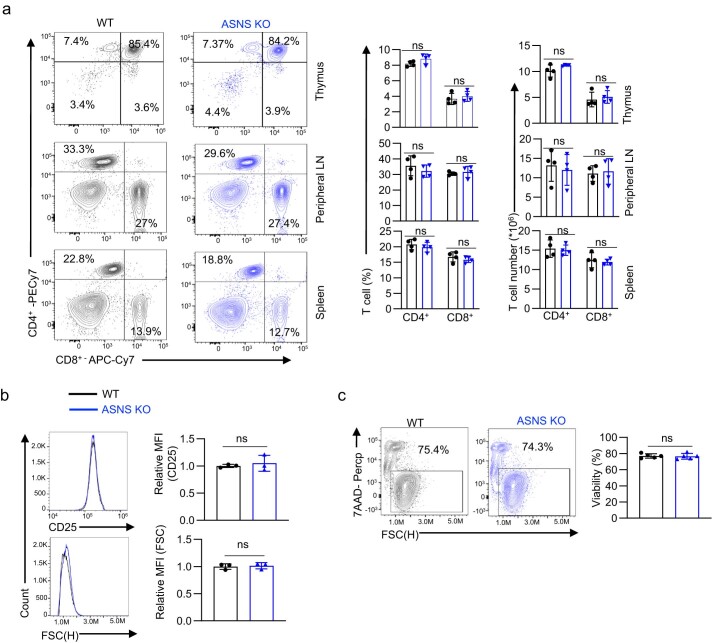
ASNS is dispensable for normal T cell development after the double-positive stage. **a**) Representative flow plots (left) and quantification of CD4^+^ and CD8^+^ distribution in the thymus, peripheral lymph nodes, and spleen (right) (n = 4 mice/group, CD4: P = 0.12 and P = 0.11; CD8^+^: P = 0.53 and P = 0.59 in thymus T cell (%) and T cell number respectively; CD4^+^: P = 0.39 and P = 0.69; CD8^+^: P = 0.56 and P = 0.77 in peripheral LN T cell (%) and T cell number respectively; CD4: P = 0.47 and P = 0.37; CD8:P = 0.77 and P = 0.66 in spleen T cell (%) and T cell number respectively. **b-c**) CD8^+^ T cells with the indicated genotype were activated, and cell surface expression of CD25, cell size (FSC) (**b**) (n = 3 experimental replicates, P = 0.59 and P = 0.76 for CD25 and cell size respectively), and cell viability (**c**) (n = 5 experimental replicates, P = 0.84) were determined by flow cytometry. Data are representative of 3 independent experiments. Error bars represent mean ± SD. ns, not significant, Statistical differences were determined by unpaired Two-tail Student’s *t-*test (**a-c**). Source data

Mammalian cells, including T cells, can engage in de novo biosynthesis of Asn through Asn synthetase (ASNS) (Fig. 2a) to replenish the intracellular Asn pool under Asn restriction^15^. Notably, Asn restriction led to a time-dependent upregulation of ASNS (Fig. 2b). To assess the role of ASNS in T cells, we generated a T cell-specific *ASNS* knockout (*ASNS* KO) mouse strain by crossing the *ASNS*^fl^ mouse strain with the CD4-Cre mouse strain. ASNS deletion did not result in any statistically significant defects in T cell development in the thymus, spleen and lymph node (Extended Data Fig. 2a). Immunoblot analyses validated the deletion of ASNS in T cells (Fig. 2c). ASNS deletion did not affect CD8^+^ T cell activation, size or viability after activation (Extended Data Fig. 2b, c). Although ASNS deletion alone did not affect CD8^+^ T_eff_ cell proliferation, it abolished CD8^+^ T_eff_ cell proliferation under Asn restriction (Fig. 2d, e). As glutamine is a vital carbon donor for replenishing the TCA cycle metabolites and aspartate biosynthesis, ASNS depletion blocked Asn de novo synthesis, as evidenced by a reduction of ^13^C_5_ and ^13^C_4_ isotopologs (from [^13^C_5_]glutamine oxidation) in glutamate and aspartate, and depletion of the ^13^C_4_ isotopolog in Asn (Fig. 2f). However, the lower level of unlabeled Asn under Asn restriction compared with the control condition suggested that ASNS-dependent de novo biosynthesis could only partially restore the intracellular Asn pool (Fig. 2f). L-asparaginase (L-ASP) has been used in clinics to treat acute lymphoblastic leukemia by depleting Asn in the blood^19^. We then assessed the homeostatic proliferation of CD8^+^ T cells in vivo following the treatment of L-ASP at a dose sufficient to deplete serum Asn and increase serum aspartate in mice without affecting glutamine and glutamate (Fig. 2g, h). In agreement with our in vitro data, L-ASP-mediated depletion of Asn significantly dampened homeostatic proliferation in adoptively transferred ASNS KO cells but not in wild-type (WT) CD8^+^ T cells (Fig. 2i–k), confirming that ASNS activity rendered T cells independent of exogenous Asn for growth in mice.

### Asn restriction rewires the central carbon metabolism

**Fig. 3:**
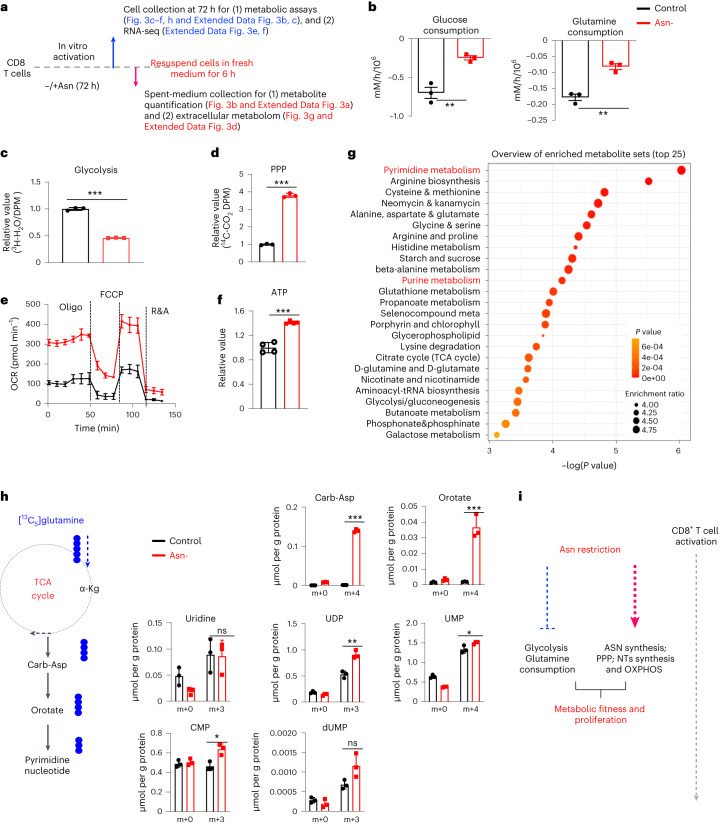
Asn restriction reduces carbon consumption but promotes nucleotide biosynthesis. **a**, Schematic diagram of sample collection and assays. **b**, The indicated metabolites were quantified by the YSI bioanalyzer. Consumption was determined by calculating the difference between blank and spent medium (6-h incubation of T cells collected in the late phase (72 h)) (*n* = 3 experimental replicates; *P* = 0.0043 and *P* = 0.002 for glucose and glutamine consumption, respectively). **c**,**d**, T cells were collected in the late phase (72 h). Glycolysis activity (**c**) (*n* = 3 experimental replicates; *P* < 0.0001) and PPP activity (**d**) (*n* = 3 experimental replicates; *P* < 0.0001) were determined by measuring ^3^H_2_O generated from D-[5-^3^H(N)]glucose and ^14^CO_2_ generated from [1-^14^C]D-glucose, respectively. **e**, OCR (in the late phase (72 h)) was determined by Seahorse (*n* = 3 experimental replicates; *P* < 0.0001), R&A, Rotenone and Antimycin A. **f**, ATP levels (in the late phase (72 h)) were determined by the CellTiter-Glo 2.0 Assay kit (*n* = 3 experimental replicates; *P* < 0.0001). Data in **b**–**f** are representative of three independent experiments. **g**, Kyoto Encyclopedia of Genes and Genomes (KEGG) enrichment analysis of the changes of extracellular metabolites (6 h spent medium). The figure is plotted with the first 25 pathways. **h**, Diagram of [^13^C_5_]glutamine catabolism through entering the downstream TCA cycle and pyrimidine biosynthesis. Filled circles denote the ^13^C label of all carbons of indicated metabolites derived from [^13^C_5_]glutamine catabolism (left panel). Metabolites in cells (in the late phase (72 h)) were analyzed by IC–UHR-FTMS (right panel); numbers on the *x* axis represent those of ^13^C atoms in given metabolites, and numbers on the *y* axis represent the levels of the metabolites (μmol per g protein). Carb-Asp, N-carbamoyl-L-aspartate; UDP, uridine diphosphate; UMP, uridine monophosphate; CMP, cytidine monophosphate; dUMP, deoxyuridine monophosphate. *n* = 3 experimental replicates; *P* < 0.0001, *P* = 0.0014, *P* = 0.9185, *P* = 0.003, *P* = 0.0418, *P* = 0.0118 and *P* = 0.0617 for Carb-Asp, orotate, uridine, UDP, UMP, CMP and dUMP, respectively. Data are representative of two independent experiments. Error bars represent mean ± s.d. *P < 0.05; **P < 0.01; ***P < 0.001. Statistical differences were determined by unpaired two-tailed Student’s *t-*test (**b**–**d**, **f** and **h**) and paired two-tailed Student’s *t-*test (**e**). MetaboAnalyst (v5.0) generated enrichment plots with a significance threshold of *P* < 0.05 (**g**). **i**, The conceptual model of Asn restriction improved T_eff_ cell metabolic fitness and proliferation by reducing carbon consumption but increasing the production of the intracellular nucleotide pool. ASN, asparagine; NTs, nucleotides; OXPHOS, oxidative phosphorylation. Source data

**Extended Data Fig. 3:**
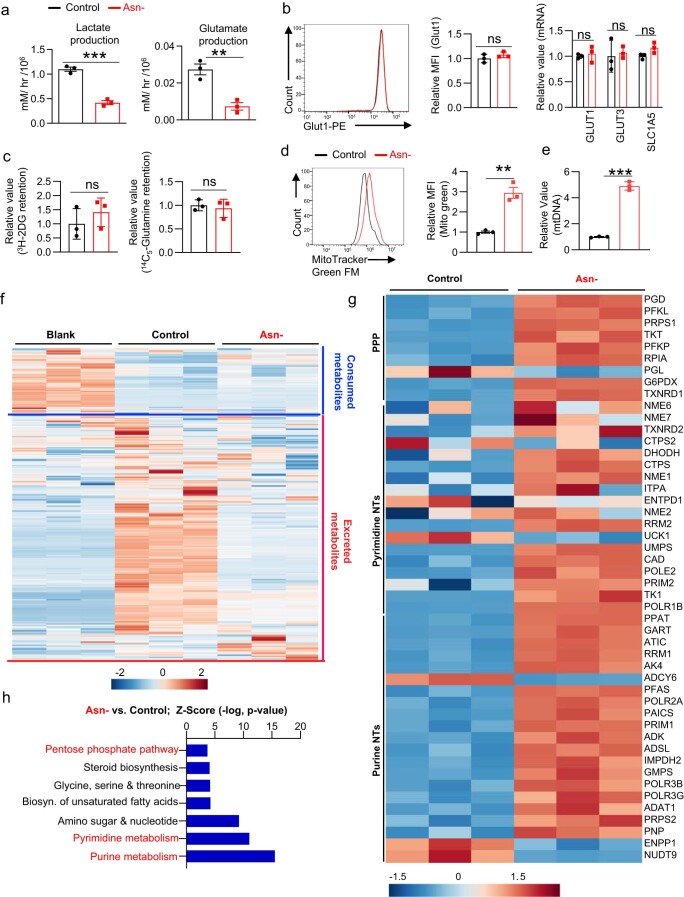
Asn restriction rewires the central carbon metabolism in CD8^+^ T cells. **a**) The indicated metabolites were quantified by the YSI bioanalyzer. The production was determined by calculating the difference between blank and spent medium (6 hr incubation of T cells collected in the late phase/72 hr) (n = 3 experimental replicates, P = 0.0003 and P = 0.0048 for lactate and glutamate production respectively). **b**) Cell surface expression of Glut1 was determined by flow, and mRNA expression of indicated genes was quantified by qPCR. (n = 3 experimental replicates, P = 0.27 for Glut1 expression and P = 0.61, P = 0.73 and P = 0.06 for Glut1, Glut 3 and Slc1a5 respectively). **c**) glucose and glutamine uptake were determined by intracellular retention of [^3^H]-2-deoxy-glucose and [^14^C_5_]-glutamine, respectively (n = 3 experimental replicates, P = 0.39 and P = 0.64 for glucose and glutamine uptake respectively). **d-e**) Mitochondria mass was determined by Mito Tracker green FM staining by flow cytometry (**d**) (n = 3, P = 0.0021), and mitochondrial DNA was quantified by qPCR (**e**) (n = 3, (P < 0.0001). Data are representative of 3 independent experiments. **f**) The extracellular metabolome of indicated groups was determined by LC-MS (Fig. 3a). (**g-h**) Differently expressed metabolic gene signatures were determined by the IPA analysis of RNAseq (in the late phase/72 h) (**g**) and a heatmap depicting log 10-fold change of differentially expressed metabolic genes (P = 0.0081) (**h**). Data from **f-h** are representative of one experiment. Error bars represent mean ± SD. *P < 0.05, **P < 0.01, *** P < 0.001. Statistical differences were determined by unpaired Two-tail Student’s *t-*test (**a-d**) paired Two-tail Student’s *t-*test (**h**). Source data

Since Asn restriction increased CD8^+^ T_eff_ cell proliferation (Fig. 1d, h), we reasoned that Asn restriction might cause metabolic changes in addition to engaging Asn de novo synthesis. We sought to determine if Asn restriction changes the central carbon metabolism to sustain robust proliferation. We took an integrated stepwise approach to comprehensively analyze the metabolic profiles of CD8^+^ T_eff_ cells under Asn restriction (Fig. 3a). The extracellular metabolome reflects the ultimate outcome of metabolic input, processing and output. We first assessed the overall consumption rate of glucose and glutamine, two primary carbon sources for proliferating cells. Unexpectedly, Asn restriction caused CD8^+^ T_eff_ cells to consume much less glucose and glutamine and excrete less lactate and glutamate (Fig. 3b and Extended Data Fig. 3a). However, Asn restriction did not affect the expression of nutrient transporters and nutrient uptake (Extended Data Fig. 3b, c). While Asn restriction reduced glycolysis, it increased glucose utilization through the PPP (Fig. 3c, d). Moreover, CD8^+^ T_eff_ cells under Asn restriction displayed a much higher oxygen consumption rate (OCR) and increased intracellular ATP production, accompanied by increased mitochondrial mass and mitochondrial DNA content (Fig. 3e, f and Extended Data Fig. 3d, e).

Next, we used a semiquantitative untargeted global metabolomics platform to profile metabolites in control (blank) media and the spent media of T cells. In parallel, we collected cells to perform RNA-seq (Fig. 3a). We classified metabolites as having changes in consumption or excretion according to whether the level in the spent media was lower or higher than in the blank media. Although Asn restriction did not change the overall consumption pattern except for glucose and glutamine, it significantly reduced metabolite excretion (Extended Data Fig. 3f). Enrichment analysis revealed that nucleotides and their precursors were among the most differentially changed metabolite groups, indicating high intracellular retention of nucleotides under Asn restriction (Fig. 3g). Since Asn restriction enhanced the PPP (Fig. 3d), we reasoned that Asn restriction might increase de novo pyrimidine nucleotide biosynthesis. To test this idea, we supplied [^13^C_5_]glutamine as a metabolic tracer in T cell culture media and followed ^13^C incorporation into downstream metabolites. The levels of corresponding ^13^C_4_ and ^13^C_3_ isotopologs of pyrimidine nucleotides and their precursors increased under Asn restriction (Fig. 3h). Moreover, these metabolic changes were accompanied by related gene expression changes in the PPP and nucleotide de novo biosynthesis, as revealed by RNA-seq (Extended Data Fig. 3g, h). Our results suggested that Asn restriction improved T_eff_ cell metabolic fitness by reducing overall carbon consumption but increasing ATP production and the intracellular nucleotide pool to enhance CD8^+^ T_eff_ cell proliferation (Fig. 3i).

### Asn restriction enhances the expression of ATF4 and NRF2

**Fig. 4:**
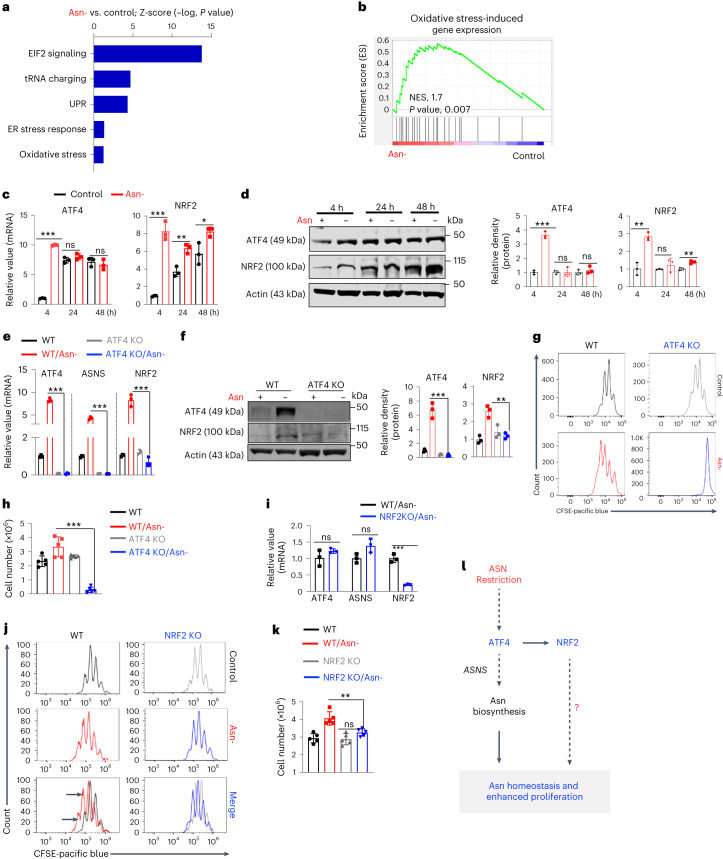
Asn restriction promotes CD8^+^ T cell proliferation by enhancing ATF4 and NRF2 expression. **a**, Differently expressed gene signatures were determined by the IPA of RNA-seq (cells collected 4 h after activation) and listed according to their z-score (*n* = 3 experimental replicates). Data are representative of one experiment. ER, endoplasmic reticulum. **b**, Oxidative stress-induced gene sets were analyzed by gene set enrichment analysis (GSEA). NES, normalized enrichment score. **c**,**d**, CD8^+^ T cells were activated in a medium with or without Asn for the indicated time. mRNA levels and protein levels of indicated molecules were determined by qPCR (**c**) (*n* = 3 experimental replicates; ATF4: *P* < 0.0001, *P* = 0.2149 and *P* = 0.4741 for 4 h, 24 h and 48 h, respectively; NRF2: *P* = 0.0003, *P* = 0.0039 and *P* = 0.0303 for 4 h, 24 h and 48 h, respectively). Immunoblot and its quantification were done by ImageJ (v1.53T) (**d**) (ATF4: *P* < 0.0001, *P* = 0.9668 and *P* = 0.4 for 4 h, 24 h and 48 h, respectively; NRF2: *P* = 0.007, *P* = 0.3771 and *P* = 0.0034 at 4 h, 24 h and 48 h, respectively). Data are representative of three independent experiments. **e**,**f**, CD8^+^ T cells with the indicated genotype and Asn status were collected 4 h after activation. The level of indicated mRNA and protein was determined by qPCR (**e**) (*n* = 3 experimental replicates; *P* < 0.0001, *P* < 0.0001 and *P* = 0.0002 for ATF4, ASNS and NRF2, respectively) and immunoblot (**f**). Immunoblot was quantified by ImageJ (v1.53T) (*P* = 0.0006 for ATF4 and *P* = 0.0029 for NRF2). Data are representative of three independent immunoblots. **g**,**h**, CD8^+^ T cells from the indicated genotype were activated in a medium with or without Asn for 72 h. Cell proliferation (**g**) was determined by CFSE staining, and the cell number was determined by a cell counter (**h**) (*n* = 5 experimental replicates; *P* < 0.0001). Data are representative of four independent experiments. **i**, The level of the indicated mRNA in cells collected 4 h after activation was determined by qPCR (*n* = 3 experimental replicates; *P* = 0.2247, *P* = 0.0583 and *P* = 0.0004 for ATF4, ASNS and NRF2, respectively). Data are representative of two independent experiments. **j**,**k**, CD8^+^ T cells from the indicated genotypes were activated in a medium with or without Asn for 72 h. Cell proliferation was determined by CFSE staining (**j**), and cell number was determined by a cell counter (**k**) (*n* = 5 experimental replicates; *P* = 0.0032). Data are representative of four independent experiments. Error bars represent mean ± s.d. *P < 0.05; **P < 0.01; ***P < 0.001. Statistical differences were determined by paired two-tailed Student’s *t-*test with a threshold of *P* < 0.05 (**a**) and unpaired two-tailed Student’s *t-*test (**c**–**f**, **h**, **i** and **k**). **l**, The conceptual model of ATF4-dependent NRF2 expression enables robust proliferation of CD8^+^ T_eff_ cells under Asn restriction. Source data

**Extended Data Fig. 4:**
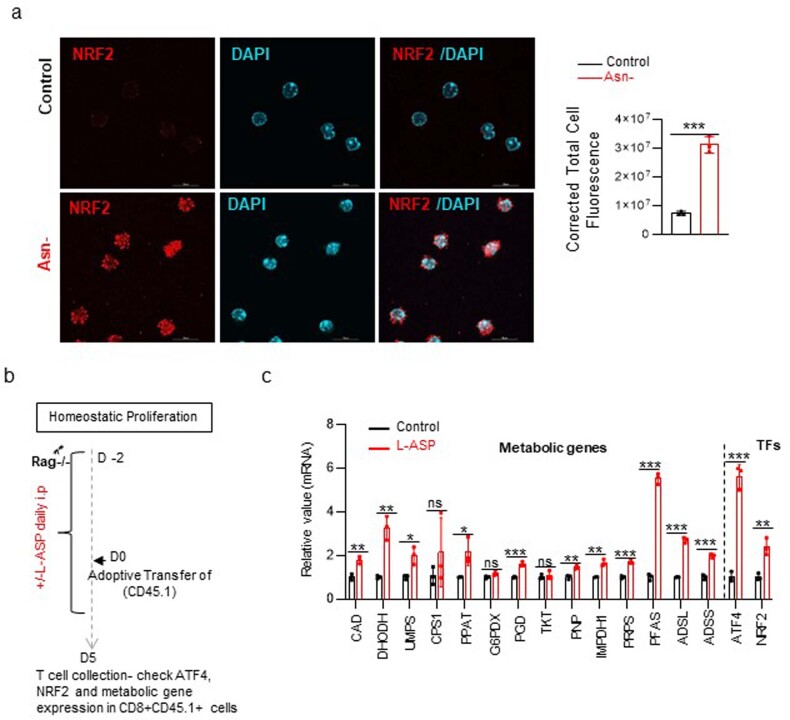
Asn restriction changes the gene expression profile in CD8^+^ T cells. **a**) Confocal imaging of NRF2 localization staining (red) and DAPI (blue) were analyzed using ImageJ (n = 3 experimental replicates, P = 0.0002). **b**) Schematic diagrams of animal experiments. **c**) The level of the indicated mRNA in cells after L-ASP was determined by qPCR (n = 3 experimental replicates, P = 0.0036, P = 0.002, P = 0.014, P = 0.31, P = 0.03, P = 0.09, P = 0.0007, P = 0.6, P = 0.003, P = 0.0015, P = 0.0006, P < 0.001, P < 0.001, P = 0.0003, P = 0002 and P = 0.0048 -CAD, DHODH, UMPS, CPS1, PPAT, G6PDX, PGD, TKT, PNP, IMPDH1, PRPs, PFAS, ADSL, ADSS, ATF4 and NRF2 respectively). Data are representative of two independent experiments. Error bars represent mean ± SD. *P < 0.05, **P < 0.01, *** P < 0.001. Statistical differences were determined unpaired Two-tail Student’s *t-*test (**a** and **c**). Source data

**Extended Data Fig. 5:**
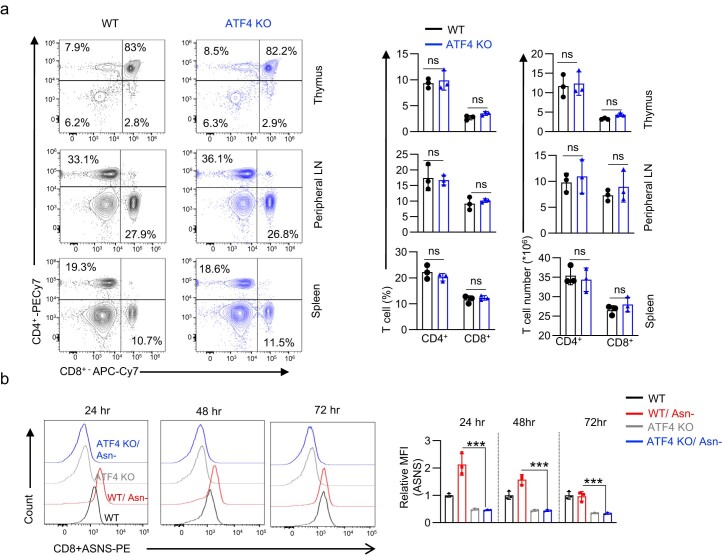
ATF4 is dispensable for T-cell development but is indispensable for ASNS induction. **a**) ATF4 is dispensable for normal T-cell development after the double-positive stage. Representative flow plots (left) and quantification of CD4^+^ and CD8^+^ distribution in the thymus, peripheral lymph nodes, and spleen (right) (n = 3 mice per group). **b**) ASNS expression in *ATF4* KO T cells was determined by flow cytometry at indicated time points (n = 3 experimental replicates, P = 0.0018, P = 0.0005 and P = 0.003 at 24, 48 and 72 h respectively). Data are representative of two experiments. Error bars represent mean ± SD. ns, not significant, Statistical differences were determined unpaired Two-tail Student’s *t-*test (**a** and **b**). Source data

Asn restriction induces activating transcription factor 4 (ATF4) in mammalian cells, which mediates an integrated stress response upon a range of environmental insults, including nutrient restriction and oxidative stress^20^. Notably, RNA-seq analysis of CD8^+^ T cells revealed that several enriched gene signatures conferred by Asn restriction are associated with ATF4 and NRF2 (Fig. 4a, b). Also, Asn restriction in CD8^+^ T cells increased the expression of both ATF4 and NRF2 as early as 4 h after activation (Fig. 4c–f), suggesting their possible involvement in regulating T_eff_ cell proliferation and inflammation upon Asn restriction. Notably, NRF2 primarily localizes in the nucleus (Extended Data Fig. 4a). Similar to the effect of Asn restriction on CD8^+^ T cells in vitro, Asn restriction via L-ASP treatment in vivo enhanced the expression of ATF4, NRF2 and related metabolic genes in adoptively transferred CD8^+^ T cells (Extended Data Fig. 4b, c). Next, we generated a T cell-specific *ATF4* knockout (*ATF4* KO) mouse strain by crossing the CD4-Cre mouse strain with the *ATF4*^fl^ mouse strain^21^. ATF4 deletion did not result in defects in T cell development in the thymus, spleen and lymph node (Extended Data Fig. 5a). In line with the previous report that ATF4 controlled the expression of ASNS and mediated the oxidative stress response, deleting ATF4 abolished the expression of ASNS and NRF2 in CD8^+^ T_eff_ cells under Asn restriction (Fig. 4e, f and Extended Data Fig. 5b). Similar to the effect of ASNS deletion on CD8^+^ T_eff_ cells (Fig. 2d, e), *ATF4* KO CD8^+^ T_eff_ cells failed to proliferate under Asn restriction (Fig. 4g, h). Since ATF4 is required for inducing NRF2 expression upon Asn restriction (Fig. 4e, f), we sought to determine the role of NRF2 in regulating CD8^+^ T_eff_ cell proliferation by using a germline *Nrf2* knockout mouse strain^22^. NRF2 deletion did not affect the expression of ASNS and ATF4 in CD8^+^ T_eff_ cells (Fig. 4i) and therefore did not abolish proliferation under Asn restriction (Fig. 4j, k). However, NRF2 deletion alleviated the hyperproliferation phenotype of CD8^+^ T_eff_ cells conferred by Asn restriction (Fig. 4j, k), supporting a model that ATF4-dependent ASNS expression replenishes the Asn pool, whereas ATF4-dependent NRF2 expression enables robust proliferation of CD8^+^ T_eff_ cells under Asn restriction (Fig. 4l).

### NRF2 is required for rewiring the central carbon metabolism

**Fig. 5:**
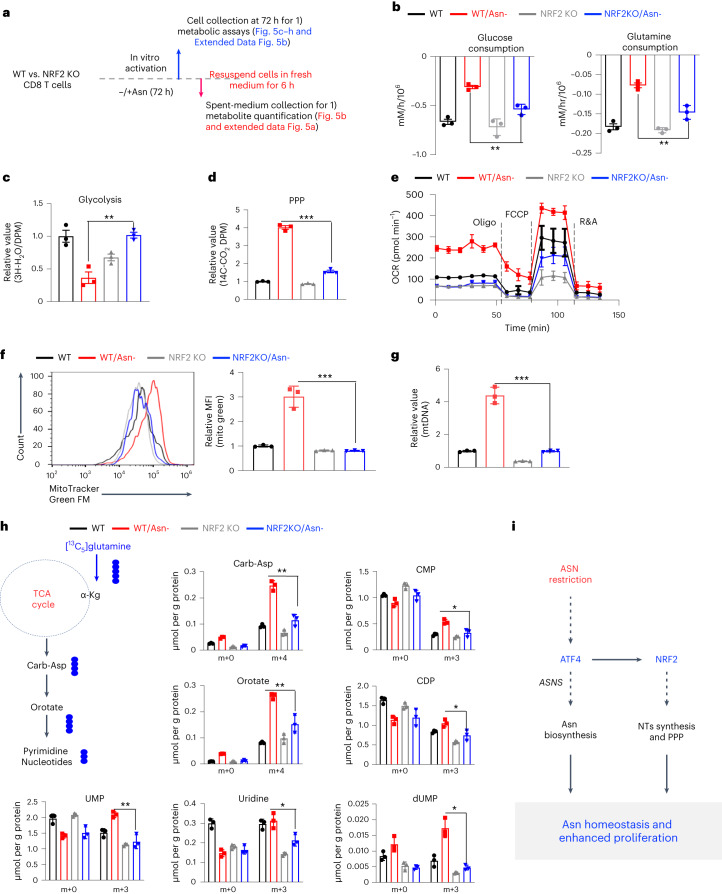
NRF2 is required to rewire the carbon metabolic program and enhance nucleotide biosynthesis under Asn restriction. **a**, Schematic diagram of sample collection and assays. **b**, The indicated metabolites were quantified by the YSI bioanalyzer. Consumption was determined by calculating the difference between blank and spent medium (6-h incubation of T cells collected in the late phase (72 h)) (*n* = 3 experimental replicates; *P* = 0.0027 and *P* = 0.0043 for glucose and glutamine, respectively). **c**,**d**, T cells were collected in the late phase (72 h). Glycolysis activity (**c**) (*n* = 3 experimental replicates; *P* = 0.0027) and PPP activity (**d**) (*n* = 3 experimental replicates; *P* < 0.0001) were determined by measuring ^3^H_2_O generated from D-[5-^3^H(N)]glucose and ^14^CO_2_ generated from [1-^14^C]D-glucose, respectively. **e**, OCR (in the late phase (72 h)) was determined by Seahorse (*n* = 3 experimental replicates). **f**,**g**, Mitochondrial mass was determined by MitoTracker Green FM staining by flow cytometry (**f**) (*n* = 3 experimental replicates; *P* = 0.0009), and mitochondrial DNA was quantified by qPCR (**g**) (*n* = 3 experimental replicates; *P* = 0.0003). Data in **b**–**g** are representative of three independent experiments. MFI, mean fluorescence intensity; mtDNA, mitochondrial DNA. **h**, Diagram of [^13^C_5_]glutamine catabolism through entering the downstream TCA cycle and pyrimidine biosynthesis. Filled circles denote the ^13^C label of all carbons of indicated metabolites derived from [^13^C_5_]glutamine catabolism (left panel). Metabolites in cells (in the late phase (72 h)) were analyzed by IC–UHR-FTMS (right panel); numbers on the *x* axis represent those of ^13^C atoms in given metabolites, and numbers on the *y* axis represent the levels of the metabolites (μmol per g protein). CDP, cytidine diphosphate. *n* = 3 experimental replicates*;* *P* = 0.0013, *P* = 0.0061, *P* = 0.0035, *P* = 0.0228, *P* = 0.0129, *P* = 0.028 and *P* = 0.0021 for Carb-Asp, orotate, UMP, uridine, CMP, CDP and dUMP, respectively. Data are representative of one experiment. Error bars represent mean ± s.d. *P < 0.05; **P < 0.01; ***P < 0.001. Statistical differences were determined by unpaired two-tailed Student’s *t-*test (**b**–**d** and **f**–**h**) and paired two-tailed Student’s *t-*test (**e**). **i**, The conceptual model of ATF4-dependent and NRF2-dependent carbon assimilation and robust effector CD8^+^ T cell proliferation. Source data

**Extended Data Fig. 6:**
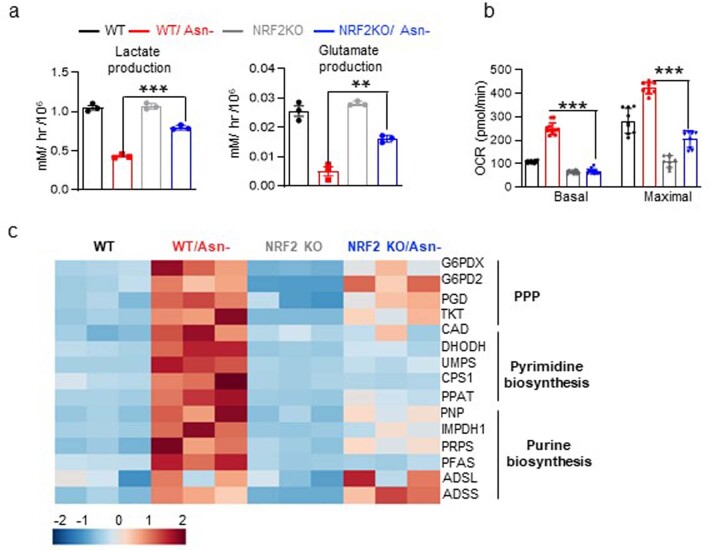
NRF2 regulates carbon consumption and nucleotide synthesis. **a**) The indicated metabolites were quantified by the YSI bioanalyzer. The production was determined by calculating the difference between blank and spent medium (6 hr incubation of T cells collected in the late phase/72 hr) (n = 3 experimental replicates, P < 0.001 and P = 0.0027 for lactate and glutamate production). **b**) OCR (in the late phase/72 hr) was determined by the Seahorse (n = 12 experimental replicates for basal and n = 9 experimental replicates for maximal OCR, P < 0.001 and P < 0.001 for basal and maximal OCR respectively). Data from **a-b** are representative of 3 independent experiments. **c**) The level of the indicated mRNA in cells collected 72 hr after activation was determined by qPCR and depicted by heatmap (log 10-fold change) (n = 3 experimental replicates). Data are representative of 2 independent experiments. Error bars represent mean ± SD. *P < 0.05, **P < 0.01, *** P < 0.001. Statistical differences were determined by unpaired Two-tail Student’s *t-*test (**a** and **b**). Source data

NRF2 is critical in maintaining redox homeostasis and regulating metabolism in physiopathological contexts, including cancer and inflammation^23^. Having shown that NRF2 is responsible for upregulating proliferation under Asn restriction (Fig. 4j, k), we sought to determine if NRF2 is required for regulating the central carbon metabolism in CD8^+^ T_eff_ cells following Asn restriction. Whereas Asn restriction significantly reduced glucose/glutamine consumption and lactate/glutamate production in WT cells, NRF2 deletion partially reversed these changes (Fig. 5a, b and Extended Data Fig. 6a). Similarly, NRF2 deletion abrogated the effects of Asn restriction on decreasing glycolysis and enhancing the PPP (Fig. 5c, d). Asn restriction could significantly increase basal and maximal respiration rates, mitochondrial mass and DNA content in WT CD8^+^ T_eff_ cells. However, NRF2 deletion in CD8^+^ T_eff_ cells reversed most of the changes caused by Asn restriction (Fig. 5e–g and Extended Data Fig. 6b), suggesting that NRF2 plays a broad role in controlling the central carbon metabolism upon Asn restriction.

Although Asn restriction reduced the overall carbon consumption from glucose and glutamine (Fig. 3b), it significantly enhanced the de novo synthesis of nucleotides to promote cell proliferation (Fig. 3h). To determine if NRF2 is required for enhancing de novo synthesis of nucleotides upon Asn restriction, we supplied [^13^C_5_]glutamine as a metabolic tracer in T cell culture media and followed ^13^C incorporation into downstream metabolites. NRF2 deletion abolished the effect of Asn restriction in increasing fractional enrichment of ^13^C isotopologs in most metabolites (Fig. 5h). In line with previous studies that NRF2 regulated the metabolic genes of the PPP and nucleotide biosynthesis in cancer cells^24^, NRF2 deletion eliminated the effect of Asn restriction on inducing many of these metabolic genes (Extended Data Fig. 6c). Collectively, our results indicated that Asn restriction rewires the central carbon metabolism and promotes nucleotide biosynthesis, thereby enhancing proliferation in CD8^+^ T_eff_ cells (Fig. 5i).

### NRF2 activation enhances CD8^+^ T cell inflammation

**Fig. 6:**
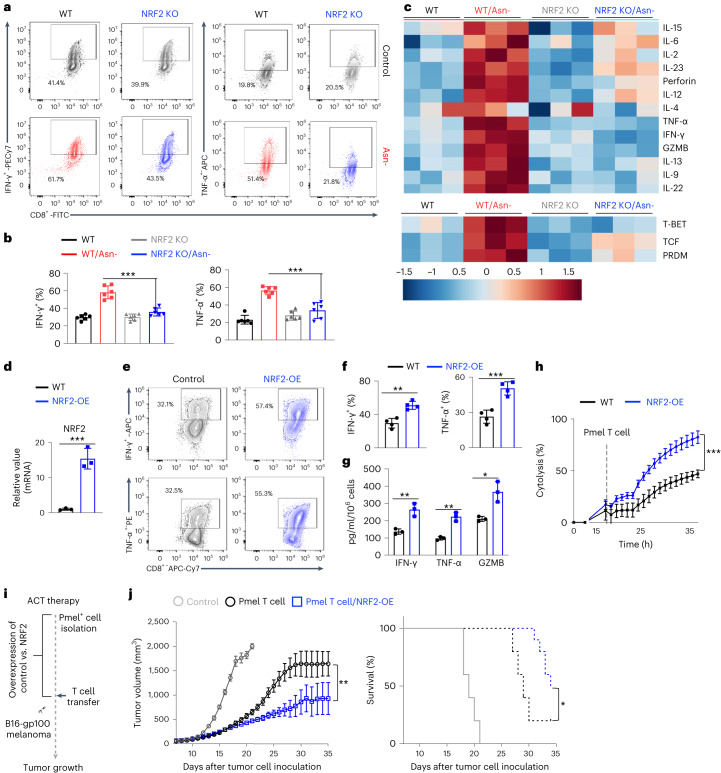
NRF2 activation enhances CD8^+^ T cell inflammation and antitumor functions. **a**,**b**, CD8^+^ T cells from the indicated genotypes were activated in a medium with or without Asn for 72 h. The expression of the indicated cytokines was determined by intracellular staining by flow cytometry (*n* = 6 experimental replicates; *P* < 0.0001 and *P* = 0.0003 for IFN-γ and TNF-α, respectively). **c**, The level of the indicated mRNA in cells collected 72 h after activation was determined by qPCR and depicted as log_10_-transformed fold change in a heatmap (*n* = 3 experimental replicates). T-bet, T-box expressed in T cells; TCF, T-cell factor; PRDM, PR domain zinc finger protein 1. Data in **a**–**c** are representative of three independent experiments. **d**–**j**, Cells with the indicated genotypes were activated for 5 days, followed by restimulation with soluble antibodies (2 days). The level of the indicated mRNA was determined by qPCR (**d**) (*n* = 3 experimental replicates; *P* = 0.0011). The expression of the indicated cytokines was determined by flow cytometry (**e**, **f**) (*n* = 3 experimental replicates for IFN-γ and *n* = 4 experimental replicates for TNF-α; *P* = 0.0013 and *P* = 0.0009 for IFN-γ and TNF-α, respectively). Indicated proteins in culture supernatants were quantified by ELISA (**g**) (*n* = 3 experimental replicates; *P* = 0.0055, *P* = 0.0013 and *P* = 0.011 for IFN-γ, TNF-α and GZMB, respectively). Cytolysis was determined by eSight (**h**) (*n* = 3 experimental replicates; *P* < 0.0001). Data in **d**–**h** are representative of three independent experiments. Error bars represent mean ± s.d. Schematic diagram of the experiment (**i**). Tumor growth (**j**, left) and Kaplan–Meier survival curves of mice bearing a B16-gp100 tumor (**j**, right) (*n* = 10 mice per group; *P* = 0.0002 for tumor growth and *P* = 0.016 for survival). Data are representative of two independent experiments. Error bars represent mean ± s.e.m. *P < 0.05; **P < 0.01; ***P < 0.001; ns, not significant. Statistical differences were determined by unpaired two-tailed Student’s *t-*test (**b**, **d** and **f**) and two-way ANOVA (**h** and **j**). Source data

Having shown that NRF2 is required to rewire central carbon metabolism to enhance CD8^+^ T_eff_ cell proliferation, we sought to determine if NRF2 is necessary to enhance inflammation under Asn restriction. Whereas Asn restriction significantly enhanced the production of proinflammatory cytokines TNF-α and IFN-γ in WT cells, NRF2 deletion reversed these changes (Fig. 6a, b). In addition, NRF2 deletion dampened the effect of Asn restriction in promoting the expression of inflammatory cytokines and transcription factors that regulate T cell effector functions (Fig. 6c). Next, we asked whether activating NRF2 could phenocopy the effects of Asn restriction in enhancing T cell effector function. We assessed the effect of NRF2 gain-of-function on Pmel^+^ CD8^+^ T_eff_ cells by overexpressing a constitutively active form of NRF2 (*NRF2*-OE) via retroviral transduction (Fig. 6d)^25^. *NRF2*-OE phenocopied the effects of Asn restriction on T cells by increasing the expression of inflammatory cytokines, enhancing cytotoxicity of tumor cells in vitro, and potentiating the antitumoral effects in vivo (Fig. 6e–j).

**Extended Data Fig. 7:**
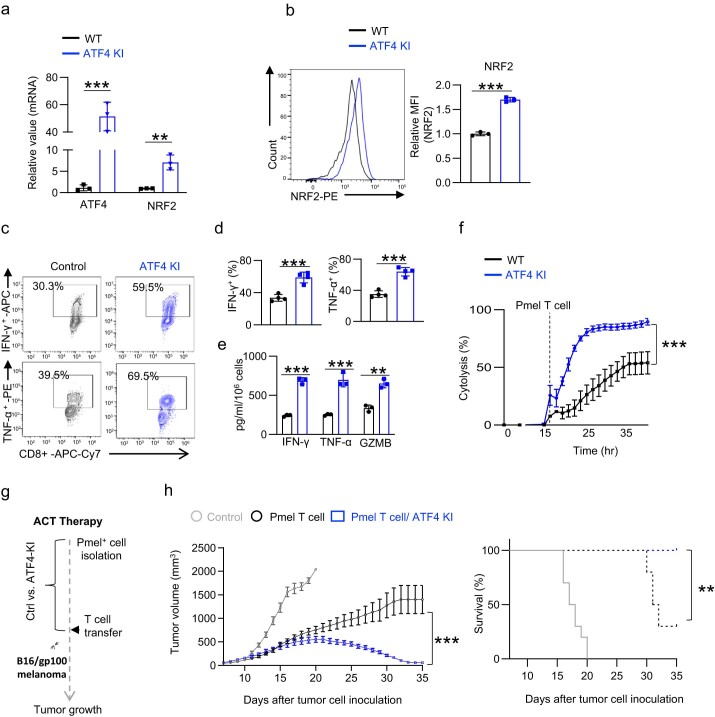
Activation of ATF4 enhances CD8^+^ T cell effector functions. **a**) The level of the indicated mRNA was determined by qPCR (n = 3 experimental replicates, P = 0.0012 and P = 0.0038 for ATF4 and NRF2 respectively). **b**) Representative flow plots (left) and quantification of NRF2 expression in indicated genotype (n = 3 experimental replicates, P < 0.001). (**c-h**) Cells with indicated genotypes were activated for 5 days, followed by restimulation with soluble antibodies (2 days). The expression of indicated cytokines was determined by flow cytometry (**c-d**) (n = 4 experimental replicates, P = 0.0007 and P = 0.0002 for IFN-γ and TNF-α respectively). The indicated protein in the cell culture medium collected 5 days after activation was quantified by ELISA (**e**) (n = 3 experimental replicates, P < 0.0001, P = 0.001 and P = 0.0014 for IFN-γ, TNF-α and GZMB respectively). Data from **a-e** are representative of 3 independent experiments. The cytolysis was determined by eSight (**f**) (n = 3 experimental replicates, P < 0.0001). Data are representative of 2 independent experiments. Error bars represent mean ± SD. Schematic diagram of the experiment (**g**). Tumor growth (left, **h**) and Kaplan-Meier survival curves (right, **h**) of mice bearing B16-gp100 tumor (n = 10 mice per group, left panel: P < 0.0001; right panel: P = 0.0013). Data are representative of 2 independent experiments. Error bars represent mean ± SEM. *P < 0.05, **P < 0.01, *** P < 0.001, ns, not significant. Statistical differences were determined by unpaired Two-tail Student’s *t-*test (**a, b** and **d-e**) and Two-way ANOVA (**f** and **h**) for tumor growth curve, and log-rank test for animal survival. Source data

Since Asn restriction induces NRF2 through ATF4 (Fig. 4e, f), we sought to determine if *ATF4* overexpression could phenocopy *NRF2*-OE on enhancing the antitumoral functions of CD8^+^ T cells. We crossed the *ATF4*^LSL-KI^ strain with the CD4-Cre strain and the Pmel transgenic strain to generate a Pmel^+^ *ATF4*-KI strain to assess the effect of *ATF4* overexpression in T cells in an antigen-specific manner. Compared with the WT control cells, Pmel^+^ *ATF4*-KI T_eff_ cells expressed a higher level of NRF2 and produced more inflammatory cytokines and effector molecules (Extended Data Fig. 7a–e). Importantly, Pmel^+^ *ATF4*-KI T_eff_ cells were more effective in killing tumor cells in vitro and controlling tumor growth in vivo (Extended Data Fig. 7f–h). Collectively, our results suggested that NRF2 plays a critical role in enhancing inflammation and antitumoral functions of CD8^+^ T_eff_ cells under Asn restriction.

### Asn restriction enhances cancer immunotherapy

**Fig. 7:**
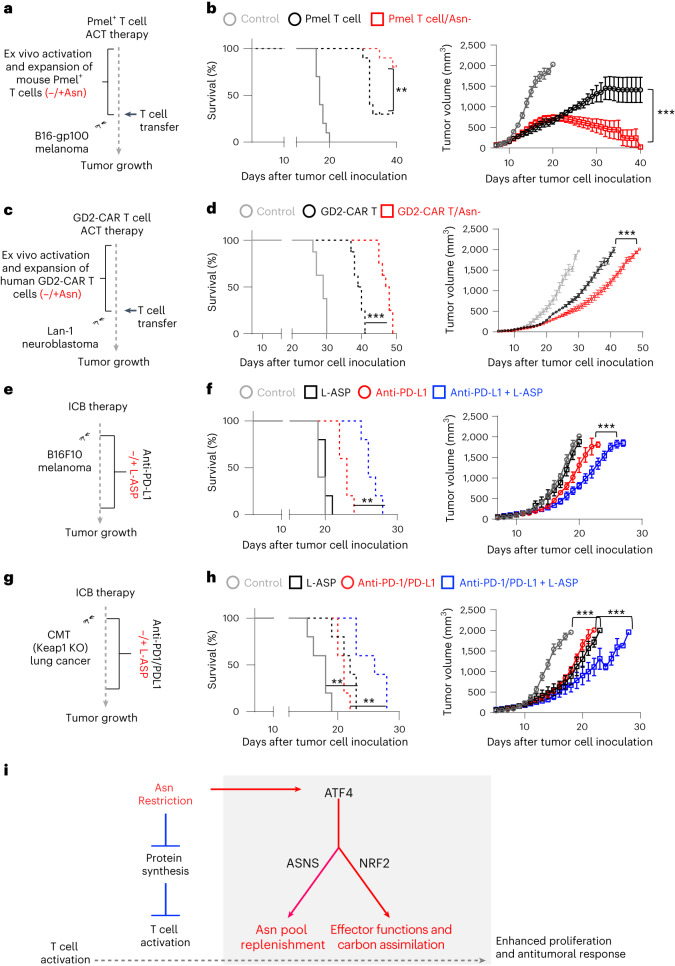
Asn restriction enhances T cell-mediated antitumor responses in vivo. **a**–**h**, Schematic diagrams of animal experiments (**a**, **c**, **e** and **g**). Kaplan–Meier survival curves and tumor growth of mice bearing a B16-gp100 tumor (**b**) (*n* = 10 mice per group; *P* = 0.0054 and *P* < 0.0001 for the left and right panels, respectively). Data are representative of three independent experiments. Kaplan–Meier survival curves and tumor growth of mice bearing a LAN-1 neuroblastoma tumor (**d**) (*n* = 8 mice per group; *P* < 0.0001 and *P* < 0.0001 for the left and right panels, respectively). Data are representative of two independent experiments. Kaplan–Meier survival curves and tumor growth of mice bearing a B16F10 melanoma tumor (**f**) (*n* = 5 mice per group; *P* = 0.0019 and *P* < 0.0001 for the left and right panels, respectively). Data are representative of three independent experiments. Kaplan–Meier survival curves and tumor growth of mice bearing a CMT *Keap-1* KO lung tumor (**h**) (*n* = 5 mice per group; *P* = 0.0085 and *P* < 0.0001 for the left and right panels, respectively). Data are representative of two independent experiments. Error bars represent mean ± s.e.m. (**b**, **d**, **f** and **h**). **P < 0.01; ***P < 0.001. Statistical differences were determined by two-way ANOVA for tumor growth curve and log-rank test for animal survival. **i**, The conceptual model of ATF4-dependent and NRF2-dependent stress signaling response, optimized carbon assimilation, and robust effector function on CD8^+^ T cells. Source data

**Extended Data Fig. 8:**
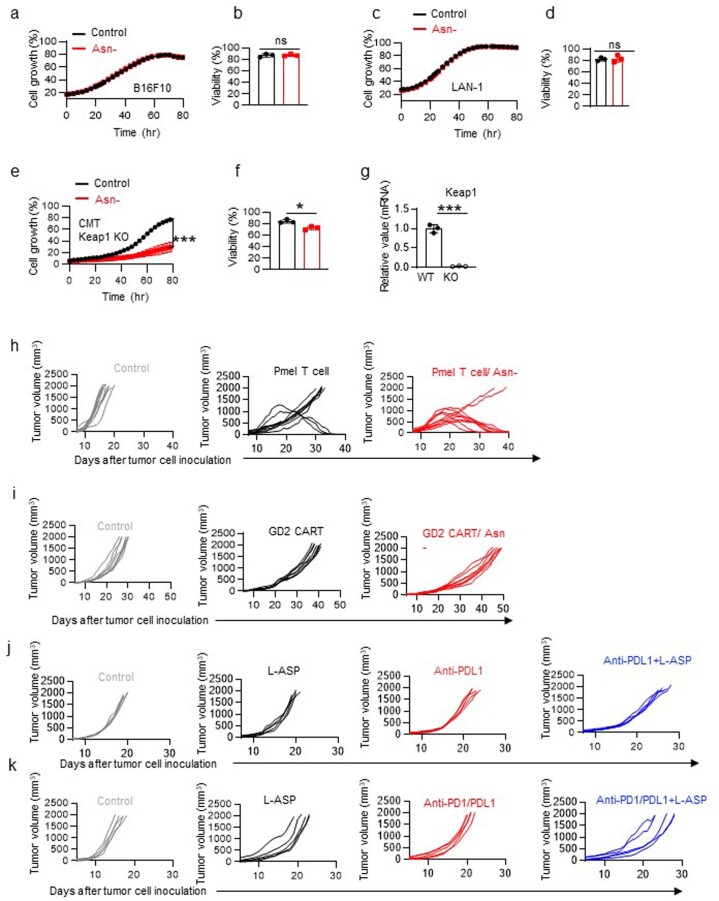
Asn restriction differentially affects T cells and tumor cells. **a-g**) B16F10, LAN-1, and CMT Keap1 KO cells were cultured in a medium with or without Asn. Cell growth **a**) (n = 3 experimental replicates), **c**) (n = 3 experimental replicates), and **e**) (n = 3 experimental replicates, P < 0.0001) was determined by live-cell imaging analysis (IncuCyte ZOOM™). Cell viability **b**) (n = 3 experimental replicates), **d**) (n = 3 experimental replicates), and **f**) (n = 3 experimental replicates, P = 0.01) was determined by 7AAD staining by flow cytometry. Data are representative of 3 independent experiments. **g**) Keap1 mRNA expression in CMT lung cancer cells was determined by qPCR (n = 3 experimental replicates, P < 0.0001). Data from **a-g** are representative of 2 independent experiments. Error bars represent mean ± SD. *P < 0.05, **P < 0.01, ns, not significant. Statistical differences were determined by unpaired Two-tail Student’s *t-*test (**b, d** and **f-g**) and Two-way ANOVA (**a, c** and **e**). **h-k**) Tumor growth of indicated groups (**h** represents Fig. 7b, i represent Fig. 7d, j represents Fig. 7f, and k represents Fig. 7h). Source data

Asn depletion therapy through L-ASP is a standard treatment for acute lymphoblastic leukemia and lymphomas in clinics because of ASNS inactivation or metabolic constraints imposed by the tumor microenvironment in these tumor types^26^. The immune checkpoint blockade (ICB) based on monoclonal antibodies targeting immune checkpoint proteins and adoptive cell transfer (ACT) based on tumor-infiltrating lymphocytes or CAR T cells are frontline cancer immunotherapies. We reasoned that Asn restriction might improve the efficacies of cancer immunotherapies by enhancing CD8^+^ T cell antitumoral activities. We used two ACT models to test this idea. We expanded Pmel T cells and GD2-CAR T cells with or without Asn in vitro, followed by transferring Pmel T cells to mice bearing B16 melanoma xenografts (Fig. 7a) and GD2-CAR T cells to immune-deficient mice bearing GD2^+^ human neuroblastoma (LAN-1) xenografts (Fig. 7c). Whereas adoptively transferred T cells alone significantly delayed tumor growth and prolonged animal survival time, expanding Pmel^+^ or GD2-CAR T cells under Asn restriction further potentiated their tumor restraining effects in these two ACT models (Fig. 7b, d and Extended Data Fig. 8h, i).

Blockade of programmed cell death protein 1 (PD-1) and programmed cell death ligand 1 (PD-L1) has been shown to elicit durable T cell-dependent antitumor responses in melanoma patients and a preclinical model of melanoma (B16 melanoma-bearing mice)^27,28^. Therefore, we assessed the antitumor effect of combining L-ASP and anti-PD-L1 antibodies in B16 melanoma-bearing mice (Fig. 7e). Notably, B16F10 and Lan-1 are Asn-prototrophic cell lines that can grow in Asn-free media in vitro (Extended Data Fig. 8a–d). In agreement with our in vitro data (Extended Data Fig. 8a, b), L-ASP-mediated depletion of Asn did not affect tumor cell growth in vivo (Fig. 7f and Extended Data Fig. 8j). However, L-ASP treatment could potentiate the antitumoral effect of anti-PD-L1 antibodies, as the combination group displayed a better outcome than the anti-PD-L1 monotherapy group (Fig. 7f and Extended Data Fig. 8j). Next, we examined the effect of combining L-ASP treatment and ICB (anti-PD-1/anti-PD-L1 antibodies) in a preclinical model of lung carcinoma (CMT167-bearing mice)^29^ (Fig. 7g). The Kelch-like ECH-associated protein 1 (*Keap-1*) gene is one of the most frequently mutated tumor suppressor genes in non-small cell lung cancer (NSCLC)^30,31^. Notably, the loss of function of *Keap-1* rendered cancer cells sensitive to NEAA (including Asn) restriction in preclinical animal models^30,31^, suggesting that combining L-ASP and ICB may be a promising strategy to treat NSCLC with *Keap-1* mutations. Therefore, we generated a *Keap-1* KO CMT167 cell line by CRISPR and confirmed that Asn restriction decreased cell proliferation and viability in vitro (Extended Data Fig. 8e–g). Whereas either L-ASP or ICB alone reduced tumor growth and prolonged animal survival time, the combinational therapy further potentiated their antitumor effects (Fig. 7h and Extended Data Fig. 8k). These results indicated that Asn restriction could be a promising and clinically relevant strategy for enhancing cancer immunotherapies against multiple cancer types.

## Discussion

In line with two recent studies^15,16^, our study showed that Asn restriction suppressed CD8^+^ T cell activation by depleting the intracellular Asn pool. Whereas one of the previous studies indicated that Asn restriction significantly inhibited CD8^+^ T cell proliferation and effector functions, the other study showed that Asn restriction failed to compromise CD8^+^ T cell proliferation, which agrees with our findings that T cells under Asn restriction could engage de novo biosynthesis to replenish the intracellular Asn pool^15,16^. Notably, both the choice of animal models and the dose of L-ASP applied to the animals are different between our work and ref. ^16^, potentially attributing to the conflicting results in the two studies^16^. In ACT experiments, Asn restriction was applied during CD8^+^ T cell expansion in vitro without further L-ASP treatment after cells were transferred into the animals. We reasoned that the observed in vivo phenotype might result from a combination of long-term and short-term effects imposed by Asn restriction. Importantly, our study revealed that the Asn restriction-induced NRF2-dependent stress signaling response confers optimized carbon assimilation and robust antitumor effector functions on CD8^+^ T cells (Fig. 7i). L-ASP is one of the first successful metabolic therapies used in clinics to treat acute lymphoblastic leukemia and lymphomas^19^. Asn-deficient media formulation can be incorporated into the current process of manufacturing CAR T cells^32^. Our study further implicates that L-ASP therapy could be integrated into ICB therapy to improve cancer patient outcomes.

CD8^+^ T cells developed a sophisticated acclimation program in response to Asn restriction. Cells switched to Asn de novo biosynthesis to replenish the intracellular Asn pool, reducing overall carbon consumption and disposal, enhancing nucleotide biosynthesis, and simultaneously eliciting robust effector functions. It is also possible that Asn coordinates with other immunomodulatory amino acids, including tryptophan and arginine, to affect T cell response^5^. Such metabolic and phenotypical changes were primarily achieved through engaging ATF4-dependent and NRF2-dependent signaling in the early phase after T cell activation. ATF4 activation increased the expression of ASNS, enabling Asn de novo biosynthesis. Asn restriction also induces NRF2 expression through ATF4. This observation aligns with a recent study suggesting that NRF2 is a direct transcriptional target of ATF4 and is induced under endoplasmic reticulum stress^33^. We observed that NRF2 primarily localizes in the nucleus. However, whether Asn restriction or ATF4 activation directly regulates the nuclear translocation of NRF2 warrants further investigation. NRF2 activation was necessary and sufficient to enhance the metabolic and functional fitness of CD8^+^ T_eff_ cells. ATF4 mediates an integrated stress response in mammalian cells to various environmental insults, including nutrient restriction and oxidative stress. Similarly, NRF2 is a critical redox sensor that transcriptionally controls the oxidative stress response in mammalian cells^34^. Recent studies have shown that NRF2 activation could enhance T cell tumor infiltration and antitumoral immune responses^35,36^. Also, ATF4 deficiency rendered CD4 T cells sensitive to oxidative stress and incapable of eliciting proper immune responses^37^. Our study suggested that NRF2 may play a crucial role in responding to changes in environmental Asn to shape CD8^+^ T cell proliferation, differentiation and functions.

Increased glycolysis and glutamine catabolism is a metabolic characteristic of activated T cells and is believed to be necessary for supporting their growth, proliferation and effector function^38,39^. However, we found that Asn restriction conferred robust proliferation and effector function to CD8^+^ T cells, accompanied by an overall decrease in glucose and glutamine consumption. By contrast, Asn restriction markedly increased oxygen consumption and mitochondrial mass in CD8^+^ T cells. Mitochondria integrate the central carbon metabolism and critical cell signaling pathways to control fate-determining processes in mammalian cells. It has been reported that CD8^+^ T_eff_ cells oxidized more glucose carbon in mitochondria in vivo than in vitro^40^. Similarly, reducing glutamine catabolism could promote oxidative phosphorylation and antitumoral activities of CD8^+^ T_eff_ cells^41^. In line with these findings, mitochondrial fitness is necessary for driving T cell proliferation, effector functions and long-term persistence in vivo^42,43^. However, the tumor microenvironment promotes T cell exhaustion and suppresses T cell-mediated antitumoral response through compromising mitochondrial biogenesis and function^44–47^. Our study suggested that CD8^+^ T_eff_ cell metabolic fitness could be enhanced by optimizing carbon assimilation via increasing mitochondrial quality and quantity, even when the overall carbon consumption is significantly reduced.

## Methods

### Mice

C57BL/6NJ, B6.129S4-*Ifng*^*tm3.1Lky*^/J, Pmel^+^(B6.Cg-*Thy1*^*a*^/CyTg(TcraTcrb)8Rest/J), *Nrf2* KO (B6.129X1-*Nfe2l2*^*tm1Ywk*^/J) and *Rag1*^−/−^ (B6.129S7-*Rag1*^*tm1Mom*^/J), B6 *Thy1.1* mice were purchased from the Jackson Laboratory. NOD.CB17-*Prkdc*^*scid*^ *IL2rg*^*tm1*^/BcgenHsd mice were purchased from Envigo. C57BL/6N-*Asns*^*tm1a(EUCOMM)Wtsi*^/H) and B6;129X1-*Gt(ROSA)26Sor*^*tm2(ATF4)Myz*^/J mice were crossed with the CD4-Cre strain to generate T cell-specific *Asns* KO and *Atf4* overexpression (*Atf4*-KI). *Atf4*-KI mice were crossed with Pmel mice to generate Pmel^+^ *Atf4* KI. *Atf4*^fl/fl^ mice were crossed with the CD4-Cre strain to generate T cell-specific *Atf4* KO^21^. Gender-matched and age-matched mice (6–12 weeks old) were used for the experiments. Both male and female WT/genetically engineered mice were used for ex vivo experiments. All mice were bred and kept in specific pathogen-free conditions at the Animal Center of the Abigail Wexner Research Institute at Nationwide Children’s Hospital. A low-fat diet (Envigo, 2920X; the irradiated form of 2020X; https://insights.envigo.com/hubfs/resources/data-sheets/2020x-datasheet-0915.pdf) was provided to the animals. The animals were euthanized by carbon dioxide asphyxiation followed by cervical dislocation under protocols approved by the Institutional Animal Care and Use Committee of the Abigail Wexner Research Institute at Nationwide Children’s Hospital (protocol number AR13-00055).

### T cell isolation and culture

Healthy donor buffy coats were obtained from the Central Ohio Region of the American Red Cross. The institutional biosafety committee of the Abigail Wexner Research Institute at Nationwide Children’s Hospital approved this research. Peripheral blood mononuclear cells (PBMCs) were isolated from buffy coats by Ficoll-Hypaque density gradient centrifugation. Isolated human mononuclear cells were stimulated with plate-bound antibodies in a medium containing human IL-2 (hIL-2). Plates were precoated with anti-human CD3 (hCD3) and anti-human CD28 (hCD28) overnight at 4 °C. To generate GD2-CAR T cells, human PBMCs were activated using plate-bound anti-hCD3 and anti-hCD28 for 2 days in the presence and absence of Asn, followed by GD2-CAR retroviral transduction and culture with hIL-2 for 8 days in the indicated medium^48^. Mouse T cells were enriched from the spleen and lymph nodes by negative selection using a mouse CD8^+^ naïve T cell isolation kit. For mouse T cell activation and proliferation assay, freshly isolated naïve CD8^+^ T cells were activated by plate-bound antibodies and cultured with mouse IL-2 (mIL-2) and mouse IL-12 (mIL-12). The culture plates were precoated with anti-mouse CD3 (mCD3) and anti-mouse CD28 (mCD28) antibodies overnight at 4 °C. For Pmel T cell activation and culture, splenocytes from Pmel transgenic mice were stimulated by human gp100 (hgp100) peptide and cultured with mIL-2 for 5 days, followed by a restimulation with mIL-2, soluble anti-mCD3, and anti-mCD28 for 2 days^49^. To analyze intercellular cytokines and effector molecules, we stimulated T cells for 4 h with anti-mCD3, anti-mCD28, brefeldin A and monensin. In some experiments, Pmel T cells (2 days after activation) were transduced with NRF2-overexpressing retrovirus and cultured for 3 days. T cells were cultured with RPMI 1640 medium supplemented with 10% fetal bovine serum (FBS), 2 mM L-glutamine, 100 units ml^−1^ penicillin, 100 μg ml^−1^ streptomycin, and 0.05 mM 2-mercaptoethanol at 37 °C and 5% CO_2_. For Asn restriction experiments, T cells were activated and cultured with Asn-free RPMI 1640 medium supplemented with 10% (v/v) dialyzed FBS (DFBS), 2 mM L-glutamine, 0.2 mM alanine, 100 units ml^−1^ penicillin, 100 μg ml^−1^ streptomycin, and 0.05 mM 2-mercaptoethanol at 37 °C and 5% CO_2_. For culturing *ATF4* KO T cells, Asn-free RPMI 1640 medium was supplemented with 10% (v/v) FBS, 2 mM L-glutamine, 100 units ml^−1^ penicillin, 100 μg ml^−1^ streptomycin, and 0.15 mM 2-mercaptoethanol as previously reported^37^. The single amino acid-deficient medium was prepared by reconstituting RPMI 1640 amino acid-free basal medium with all but one amino acid (the corresponding amino acid). The amino acid-proficient control medium was supplemented with 200 μM alanine, as switching FBS to DFBS abolishes the main source of alanine in the T cell medium^50^. DFBS was made by dialyzing FBS against 100 volumes of distilled water (changed five times in 3 days) using dialysis cassettes (2K MWCO) at 4 °C. Additional information on the concentrations of antibodies, cytokines and resources is listed in Supplementary Tables 1 and 2.

### Tumor cell line culture

B16F10 was acquired from the American Type Culture Collection (ATCC) (CRL-6475). The LAN-1, B16-gp100 and CMT167 cell lines were provided by X. Song, N. Restifo and W. Terence, respectively. Tumor cell lines were cultured with regular RPMI 1640 or Asn-free RPMI 1640 medium supplemented with 10% (v/v) DFBS, 2 mM L-glutamine, 100 units ml^−1^ penicillin, and 100 μg ml^−1^ streptomycin at 37 °C and 5% CO_2_. The CMT *Keap1* KO cell line was generated by CRISPR by isolating a monoclonal cell population by limiting dilution.

### ACT for tumor immunotherapy

For the B16 melanoma ACT model, female C57BL/6 mice were implanted subcutaneously with 1 × 10^5^ cells of B16-gp100 in the flank, followed by sublethal irradiation (600 cGy) and intravenous injection of 1 × 10^6^ Pmel T cells on day 7. For the LAN-1 xenograft, male severe combined immunodeficient (SCID) mice were implanted subcutaneously with 1.5 × 10^6^ LAN-1 tumor cells in the flank, followed by intravenous injection of 7 × 10^6^ live GD2-CAR T cells without removing dead cells. GD2-CAR T cells were generated in the presence and absence of Asn and expanded for 10 days. Before ACT, cell viability (more than 65%) and cytokine production were measured to ensure the quality of donor cells. For the B16F10 melanoma model, female C57BL/6 mice were implanted subcutaneously with 1 × 10^5^ cells of B16-gp100 in the flank, followed by intraperitoneal injection of anti-PD-L1 antibody (twice per week). For the CMT *Keap-1* KO lung cancer model, female C57BL/6 mice were implanted subcutaneously with 1 × 10^5^ cells in the flank, followed by intraperitoneal injection of anti-PD-1 and anti-PD-L1 antibodies (three times per week). In LAN-1 xenograft experiments, male and female mice were used as recipient hosts for the implantation of tumor cells. Since both cell lines are derived from female mice, female hosts were used for B16 and CMT allograft experiments. In some experiments, L-ASP was intraperitoneally administered every day. In all experiments, tumor sizes were measured daily, and tumor volume was calculated by length × width^2^ × π/6. For survival studies, animals were monitored daily until one of the following end points was reached: (1) tumor diameter reaches 2 cm, (2) tumor ulceration reaches 1 cm in diameter or has persistent bleeding, (3) animals lose over 20% of body weight, or (4) animals experience breathing difficulty or poor mobility.

### ACT for homeostatic proliferation

For homeostatic proliferation in lymphopenic *Rag*^*−/−*^ mice, naïve CD8^+^ T cells isolated from donor mice were mixed with WT and KO cells at a 1:1 ratio and labeled with carboxyfluorescein diacetate succinimidyl ester (CFSE). Approximately 1 × 10^7^ cells in 100 μl of PBS were intravenously injected into 6–8-week-old gender-matched host mice, which were then treated with L-ASP for 3 days. Mice were euthanized after 4 days, and lymph nodes and spleen were collected and processed to assess cell ratio and proliferation by flow cytometry analysis. Serum was collected from control and L-ASP-treated mice, and metabolites in serum were quantified by gas chromatography–mass spectrometry (GC–MS).

### Flow cytometry

For analyzing surface markers, cells were stained in PBS containing 2% (w/v) BSA and the indicated antibodies. In some experiments, T cells were stimulated by the cell stimulation cocktail (4 h at 37 °C), followed by staining with antibodies for cell-surface protein. Then, cells were fixed and permeabilized by FoxP3 fixation/permeabilization solution, followed by staining with antibodies for intracellular proteins. Cell proliferation was determined by CFSE staining, and cell viability was evaluated by 7-aminoactinomycin D (7-AAD) staining. For analyzing protein content, cells were incubated with O-propargyl-puromycin (OPP; 1 h at 37 °C), followed by fixation with FoxP3 permeabilization solution and 5 FAM-Azide stainings (0.5 h at 37 °C) according to the protein synthesis assay kit. For analyzing the cell cycle profile, cells were incubated with bromodeoxyuridine (BrdU; 1 h at 37 °C), followed by cell surface staining, fixation and permeabilization according to the Phase-Flow Alexa Fluor 647 BrdU Kit. Flow cytometry data were acquired on NovoCyte and were analyzed with FlowJo software. Additional information on flow cytometry antibodies, dilution and resources is listed in Supplementary Table 1. Gating strategies are shown in Supplementary Figs. 1 and 2.

### Real-time cytotoxicity assay

The target tumor cells (B16-gp100 mouse melanoma or LAN-1 human neuroblastoma cells) were used for determining the cytotoxic potential of Pmel T cells or GD2-CAR T cells using xCELLigence RTCA eSight. A 50-μl medium was added to E-Plates 96 for measurement of background values. Target cells were seeded in an additional 50-μl medium at a density of 3,000 cells per well for B16-gp100 and 6,000 cells per well for LAN-1 and cultured for 16–18 h before adding T cells at an effector:target ratio of 5:1. Cell density and cell ratio were determined by previous titration experiments. Impedance-based measurements of the normalized cell index were performed every 15 min upon T cell addition. Cell index values were exported, and the percentage of lysis was calculated using RTCA Software Pro.

### Quantification of cytokines in the cell culture supernatant

Pmel T cells were activated with hgp100 peptide for 4 days, followed by restimulation with anti-mCD3 and anti-mCD28 antibodies for 48 h. Cells were resuspended in fresh medium at a density of 2 × 10^6^ cells ml^−1^ and cultured for 6 h. Cytokine production in the cell culture supernatant was determined by enzyme-linked immunosorbent assay (ELISA). GD2-CAR T cells were resuspended in fresh medium at a density of 2 × 10^6^ cells ml^−1^ and cultured for 6 h. Cytokine production in the cell culture supernatant was determined by LEGENDplex Human CD8/NK Panel according to the manufacturer’s instructions. The data were collected by NovoCyte and analyzed using LEGENDplex Data Analysis Software.

### Quantification of metabolites in the cell culture supernatant

The naïve CD8^+^ T cells were activated by plate-bound anti-mCD3 and anti-mCD28 antibodies for 72 h. Cells were resuspended in the indicated medium at a density of 2 × 10^6^ cells ml^−1^ for 6 h. The levels of glucose, lactate, glutamine and glutamate in blank medium (without cells) and spent medium were measured by the bioanalyzer (YSI 2900). The metabolite consumption and production were determined by calculating the difference between blank and spent medium. In some experiments, the arginine level was determined by the L-Arginine Assay Kit.

### Radioactive tracer-based metabolic assays

The radioactive tracer-based metabolic assay was performed as described previously^51^. Glycolysis was measured by the generation of ^3^H_2_O from [5-^3^H(N)]D-glucose, the PPP was measured by the generation of ^14^CO_2_ from [1-^14^C]D-glucose, and glutamine oxidation activity was measured by the generation of ^14^CO_2_ from [U-^14^C]glutamine. For assays generating ^14^CO_2_, 5 × 10^6^ T cells in 0.5 ml of fresh medium were dispensed into 7-ml glass vials with a PCR tube containing 50 μl of 0.2 N NaOH glued on the sidewall. After adding 1 μCi radioactive tracer, the vials were capped using a screw cap with a rubber septum and incubated for 2 h at 37 °C. The assay was then stopped by injecting 100 μl of 5 N HCl into the vial. Vials were kept at 18–22 °C overnight to trap the ^14^CO_2_. The NaOH solution in the PCR tube was then transferred to scintillation vials containing 10 ml of scintillation solution for counting. A cell-free sample containing the same amount of tracer was included as a background control. For assays generating ^3^H_2_O, 1 μCi radioactive tracer was added to the suspension of one million cells in 0.5 ml of fresh medium in 48 wells, then incubated for 2 h at 37 °C. The assay was stopped by transferring samples to a 1.5-ml microcentrifuge tube containing 50 μl of 5 N HCl, which was placed in a 20-ml scintillation vial containing 0.5 ml of water. Then, the vial was capped and sealed. ^3^H_2_O was separated from other radiolabeled metabolites overnight at room temperature by evaporation diffusion. The 1.5-ml microcentrifuge tube was removed, and 10 ml of scintillation solution were added to the vial before counting. A cell-free sample containing 1 μCi radioactive tracer was included as a background control. Radioactivity was measured by liquid scintillation counting.

### Stable isotope labeling

Experiments in Figs. 2f and 3h: Naïve CD8^+^ T cells were activated by plate-bound anti-mCD3 and anti-mCD28 antibodies for 36 h. Then, cells were reseeded at a density of 2 × 10^6^ cells ml^−1^ in a conditional medium containing 4 mM [^13^C_5_]glutamine for 24 h. Samples were collected and washed three times with PBS before being snap-frozen.

Experiments in Fig. 5h: Naïve CD8^+^ T cells were activated by plate-bound anti-mCD3 and anti-mCD28 antibodies for 72 h. Then, cells were reseeded at the density of 2 × 10^6^ cells ml^−1^ in a conditional medium containing 4 mM [^13^C_5_]glutamine for 6 h. Samples were collected and washed three times with PBS before being snap-frozen.

### Gas chromatography–mass spectrometry (GC–MS)-based analysis

GC–MS was performed as previously described^52^. Cell pellets were resuspended in 0.45 ml of −20 °C methanol/water (1:1 v/v) containing 20 µM L-norvaline as the internal standard. Further extraction was performed by adding 0.225 ml of chloroform, vortexing, and centrifuging at 15,000 × *g* for 5 min at 4 °C. The upper aqueous phase was evaporated under a vacuum using a SpeedVac centrifugal evaporator. Separate tubes containing varying amounts of standards were evaporated. Dried samples and standards were dissolved in 30 μl of isobutylhydroxylamine hydrochloride in pyridine and incubated for 20 min at 80 °C. An equal volume of *N*-*tert*-butyldimethylsilyl-*N*-methyltrifluoroacetamide (MTBSTFA) was added and incubated for 60 min at 80 °C. After derivatization, samples and standards were analyzed by GC–MS using a Rxi-5ms column (15 m × 0.25 i.d. × 0.25 μM; Restek) installed in a Shimadzu GCMS-QP2010 Plus. The GCMS-QP2010 Plus was programmed with an injection temperature of 250 °C, 1.0 µl injection volume, and a split ratio of 1/10. The GC oven temperature was initially 130 °C for 4 min, rising to 250 °C at 6 °C min^−1^ and to 280 °C at 60 °C min^−1^, with a final hold at this temperature for 2 min. GC flow rate, with helium as the carrier gas, was 50 cm s^−1^. The GC–MS interface temperature was 300 °C, and (electron impact) ion source temperature was 200 °C, with 70 eV ionization voltage. Fractional labeling from ^13^C substrates and mass isotopomer distributions were calculated as described^52^. Data from standards were used to construct standard curves in MetaQuant^53^, from which metabolite amounts in samples were calculated. Metabolite amounts were corrected for recovery of the internal standard and for ^13^C labeling to yield total (labeled and unlabeled) quantities in nmol per sample and then adjusted to protein.

### Ion chromatography–ultra-high-resolution Fourier transform mass spectrometry (IC–UHR-FTMS)-based analysis

The frozen cell pellets were homogenized in 60% cold CH_3_CN in a ball mill for denaturing proteins and optimizing extraction. Polar metabolites were extracted by the solvent partitioning method with a final CH_3_CN:H_2_O:CHCl_3_ ratio of 2:1.5:1 (v/v), as described previously^54^. The polar extracts were reconstituted in Nanopure water before analysis on a Dionex ICS-5000+ ion chromatography system interfaced with an Orbitrap Fusion Tribrid mass spectrometer (Thermo Scientific) as previously described^55^ using an *m*/*z* scan range of 80–700. Peak areas were integrated and exported to Microsoft Excel via TraceFinder software (v3.3; Thermo Fisher Scientific) before natural abundance correction^56^. The isotopolog distributions of metabolites were calculated as the mole fractions as previously described^57^. The number of moles of each metabolite was determined by calibrating the natural abundance-corrected signal against authentic external standards. The amount was normalized to the amount of extracted protein and is reported in µmol per g protein.

### Liquid chromatography–mass spectrometry (LC–MS)-based analysis

As described previously, sample preparation and analysis were conducted at Metabolon^58^. In brief, sample preparation involved protein precipitation and removal with methanol, shaking and centrifugation. The resulting extracts were profiled on an accurate mass global metabolomics platform consisting of multiple arms differing by chromatography methods and mass spectrometry ionization modes to achieve broad coverage of compounds differing in physiochemical properties such as mass, charge, chromatographic separation and ionization behavior. Metabolites were identified by automated comparison of the ion features in the experimental samples to a reference library of chemical standard entries that included retention time, molecular weight (*m/z*), preferred adducts, and in-source fragments, as well as associated MS spectra, and were curated by visual inspection for quality control using software developed at Metabolon. MetaboAnalyst was used to range-scale data and provide pathway analysis of metabolites significantly changed (*P* < 0.05).

### OCR

OCR was determined by the Seahorse XFe96 Analyzer according to the manufacturer’s instructions. In brief, naïve CD8^+^ T cells were activated by plate-bound anti-mCD3 and anti-mCD28 antibodies for 72 h. Then, 1 × 10^5^ cells were collected, washed by PBS, and suspended in 50 µl of assay medium (Seahorse XF RPMI Assay Medium) containing 10 mM glucose, 2 mM glutamine and 1 mM pyruvate, then seeded in each well in a poly-D-Lysine precoated XF96 Cell Culture Microplate. Then, the plate was centrifuged at 200 × *g* for 2 min in a zero-braking setting to immobilize cells, followed by the addition of 130 µl of assay medium and incubation in a non-CO_2_ incubator for 30 min. OCR was measured under basal conditions and in response to oligomycin, carbonyl cyanide-*p*-trifluoromethoxyphenylhydrazone (FCCP), or 100 nM rotenone with 1 µM antimycin A. Data analysis was performed using the Seahorse Wave software.

### Measurement of ATP levels

ATP levels were measured using the CellTiter-Glo 2.0 Assay kit according to the manufacturer’s instructions. In brief, 1 × 10^5^ cells were suspended with 50 ul of PBS in 96-well white-walled tissue culture plates, and an equal volume of CellTiter-Glo 2.0 reagent was added to each well. The plate was put on a shaker for gentle mixing and incubated for 30 min at room temperature. Luminescence was measured by the Microplate Reader.

### Glucose and glutamine uptake assay

Mouse CD8^+^ T cells were stimulated with plate-bound anti-CD3 and anti-CD28 antibodies for 3 days. Then, 1 × 10^7^ active T cells were suspended in 600 μl of media containing 11 mM cold glucose and 4 mM glutamine. We added 3 μCi [^3^H]2-deoxyglucose or 1 μCi [^14^C_5_]glutamine into the media and incubated them for 10 min at 37 °C. Cell suspension aliquots of 200 μl were loaded onto separation buffer layers, prepared by adding 100 μl of 20% perchloric acid/8% sucrose solution to the bottom of a 1.5-ml microcentrifuge tube and overlaid with 800 μl of 1-bromododecane. The tubes containing cell suspension and separation buffer layers were centrifuged at 12,000 × *g* for 1 min. The tube was then snap-frozen in a dry-ice bath, and the tip of the tube (bottom layer with cell lysis) just above the perchloric acid/sucrose/bromododecane interface was cut off and transferred into a well of a 24-well plate. The tip was washed with 300 μl of 0.5% SDS/1% Triton X-100. The solution collected from each tube was then transferred to a scintillation vial containing 10 ml of scintillation solution for counting.

### NRF2 localization by confocal microscopy

Mouse CD8^+^ T cells were stimulated with plate-bound anti-CD3 and anti-CD28 antibodies for 4 h, and cells were resuspended in fresh medium and incubated with poly-L-lysine coated coverslips in 6-well cell culture plates for 1 h at 37 °C. These cells were fixed by 4% paraformaldehyde and incubated for 15 min at room temperature, followed by three PBS washes. After fixation, cells were permeabilized with 0.1% Triton for 5 min, washed extensively with PBS, and blocked with cold blocking buffer (1x PBS, 0.5% standard grade BSA) for 30 min on ice. The blocking buffer was carefully removed and incubated with NRF2 antibody or isotype control overnight at 4 °C. Cells were incubated with a secondary antibody (Alexa Fluor 647) for 1 h on ice in the dark, followed by three PBS washes. Cells were counterstained with 4,6-diamidino-2-phenylindole (DAPI), and the coverslips were mounted with an antifading mounting medium. The images were recorded on a Nikon AX R confocal microscope. For quantification, fluorescence intensity was quantified by ImageJ software, and corrected total cell fluorescence (CTCF) was calculated by using the following equation: CTCF = integrated density (of the selected cell) − (area of the selected cell × mean fluorescence of the background).

### Western blot analysis, RNA extraction, qPCR, and RNA-seq analysis

Details are provided in the Supplementary Information and primer sequences are provided in Supplementary Table S3.

### Statistical analysis

Statistical analysis was conducted using the GraphPad Prism software (v8.0.1). *P* values were calculated with two-way analysis of variance (ANOVA) for in vivo experiments. Kaplan–Meier curves and the corresponding log-rank test was used to evaluate the statistical differences between groups in survival studies. Paired and unpaired two-tailed Student’s *t*-tests were used to assess differences in other experiments. The R programming language software was used for Metabolon (v5.0) and RNA-seq data analysis. *P* values less than 0.05 were considered significant, with *P* < 0.05, *P* < 0.01 and *P* < 0.001 indicated as *, ** and ***, respectively; ns indicated no significant differences.

### Reporting summary

Further information on research design is available in the Nature Portfolio Reporting Summary linked to this article.

## Supplementary information

Supplementary Information Methods, Supplementary Tables 1–3 and Supplementary Figs. 1 and 2.

Reporting Summary

## Data Availability

The RNA-seq data sets generated for this study are in the Gene Expression Omnibus (GEO) under accession number GSE201870. The authors declare that all other data supporting the findings of this study are available within the paper and supplementary information files. Source data are provided with this paper.

## Source data

Source Data Fig. 1 Statistical source data.
Source Data Fig. 2 Statistical source data and unprocessed western blots.
Source Data Fig. 3 Statistical source data.
Source Data Fig. 4 Statistical source data and unprocessed western blots.
Source Data Fig. 5 Statistical source data.
Source Data Fig. 6 Statistical source data.
Source Data Fig. 7 Statistical source data.
Source Data Extended Data Fig. 1 Statistical source data.
Source Data Extended Data Fig. 2 Statistical source data.
Source Data Extended Data Fig. 3 Statistical source data.
Source Data Extended Data Fig. 4 Statistical source data.
Source Data Extended Data Fig. 4a Unprocessed confocal images.
Source Data Extended Data Fig. 5 Statistical source data.
Source Data Extended Data Fig. 6 Statistical source data.
Source Data Extended Data Fig. 7 Statistical source data.
Source Data Extended Data Fig. 8 Statistical source data.
