# Supplementary material for: Asparagine restriction enhances CD8+ T cell metabolic fitness and antitumoral functionality through an NRF2-dependent stress response
Source: Nat Metab. 2023 Aug 7;5(8):1423–39. doi: 10.1038/s42255-023-00856-1 (PMC10447245; doi:10.1038/s42255-023-00856-1)
Supplement: Supplementary file 1 — Methods, Supplementary Tables 1–3 and Supplementary Figs. 1 and 2. [file 42255_2023_856_MOESM1_ESM.pdf]

nature metabolism

Article

https://doi.org/10.1038/s42255-023-00856-1

Asparagine restriction enhances CD8^{+} T cell metabolic fitness and antitumoral functionality through an NRF2-dependent stress response

In the format provided by the authors and unedited

Supplementary Information

Methods:

Western blot analysis

Cells were harvested, lysed, and sonicated at 4 ℃ in a lysis buffer (50 mM Tris-HCl, pH 7.4, 150 mM NaCl, 0.5% SDS, 5 mM sodium pyrophosphate, protease, and phosphatase inhibitor tablet). Cell lysates were centrifuged at 13,000 × g for 15 min to collect the supernatant. The protein concentrations were determined by the Pierce™ BCA Protein Assay kit. The samples were boiled in NuPAGE® LDS Sample Buffer and Reducing solution for 5 min. The proteins were separated by NuPAGE Protein Gels, transferred to PVDF membranes by the iBlot Gel Transfer Device, then incubated with primary antibodies followed by a corresponding secondary antibody. Immunoblots were developed with LI-COR. Detailed information for primary antibodies and secondary antibodies was provided in Supplementary Table S1.

RNA extraction, qPCR, and RNAseq

Total RNA was isolated using the Quick-RNATM MiniPrep Kit and was reverse transcribed using random hexamer and M-MLV Reverse Transcriptase. BIO-RAD CFX284^{TM} Real-Time PCR Detection System was used for SYBR green-based quantitative PCR. The relative gene expression was determined by the comparative *CT* method, also referred to as the $2^{-\Delta\Delta CT}$ method. The data were presented as the fold change in gene expression normalized to an internal reference gene (beta2-microglobulin) relative to the control (the first sample in the group). Fold change = $2^{-\Delta\Delta C}{}_{T}$ = $[(CT_{\text{gene of interest}} - CT_{\text{internal reference}})]$ sample A - $[(CT_{\text{gene of interest}} - CT_{\text{internal reference}})]$ sample B. Samples for each experimental condition were run in triplicated PCR reactions. Primer sequences were obtained from Primer Bank to detect target genes (Supplementary Table S3).

For RNA sequencing analysis, total RNA was extracted using RNeasy Mini Kit and treated with DNase I according to the manufacturer's instructions. After assessing the quality of total RNA using an Agilent 2100 Bioanalyzer and RNA Nanochip, 150 ng total RNA was treated to deplete ribosomal RNA (rRNA) using target-specific oligos combined with rRNA removal beads. Following rRNA removal, mRNA was fragmented and converted into double-stranded cDNA. Adaptor-ligated cDNA was amplified by limit cycle PCR. After library quality was determined via Agilent 4200 TapeStation and quantified by KAPA qPCR, approximately 60 million paired-

end 150 bp sequence reads were generated on the Illumina HiSeq 4000 platform. Quality control and adapter trimming were accomplished using the FastQC (version 0.11.3) and Trim Galore (version 0.4.0) software packages. Trimmed reads were mapped to the Genome Reference Consortium GRCm38 (mm10) murine genome assembly using TopHat2 (version 2.1.0), and feature counts were generated using HTSeq (version 0.6.1). Statistical analysis for differential expression was performed using the DESeq2 package (version 1.16.1) in R, with the default Benjamini-Hochberg *p-value* adjustment method. The Ingenuity Pathway Analysis (IPA) software, the Gene Set Enrichment Analysis (GSEA) software, and the R Programming Language software were used to analyze gene signature and pathway enrichment.

Supplementary table S1. List of antibodies and cytokines

Antibodies and recombinant proteins	Vendor	Catalog #	Application
APC anti-mouse IFN-γ Antibody	BioLegend	505810	1:200 dilution/Flow cytometry
PE/Cyanine7 anti-mouse IFN-γ Antibody	BioLegend	505826	1:200 dilution/Flow cytometry
APC Anti-mouse TNF-α	BioLegend	506308	1:200 dilution/Flow cytometry
APC/Cyanine7 anti-mouse CD8a Antibody	BioLegend	100714	1:300 dilution/Flow cytometry
APC anti-mouse TCR β chain Antibody	BioLegend	109211	1:100 dilution/Flow cytometry
PE NRF2 rabbit monoclonal Antibody	Cell signaling	14409	1:100 dilution/Flow cytometry
APC/Cyanine7 anti-mouse thy1.1 Antibody	BioLegend	202520	1:100 dilution/Flow cytometry
Percp anti-mouse thy1.2 Antibody	BioLegend	140316	1:100 dilution/Flow cytometry
CD25 Monoclonal Antibody (PC61.5), PE	eBioscience	12-0251	1:300 dilution/Flow cytometry
FITC anti-mouse CD8 Antibody	BioLegend	100705	1:200 dilution/Flow cytometry
APC/Cyanine7 anti-human CD8a Antibody	BioLegend	300926	1:200 dilution/Flow cytometry
PE/Cyanine7 anti-mouse CD4	BioLegend	100422	1:200 dilution/Flow cytometry
PE/Cyanine7 anti-human IFN-γ Antibody	BioLegend	506518	1:200 dilution/Flow cytometry
PE anti-human TNF-α Antibody	BioLegend	502909	1:200 dilution/Flow cytometry
InVivoMAb anti m PD-L1	BioXcell	BE0101	200 µg per dose / invivo immunotherapy
InVivoMAb anti m PD-1	BioXcell	BE0146	200 µg per dose / invivo immunotherapy
InVivoMAb Rat IgG2b Isotype control	BioXcell	BE0090	200 µg per dose / invivo immunotherapy
InVivoMAb anti-mouse CD3	BioXcell	BE0001	2-5 µg/ml / T cell activation
InVivoMAb anti-mouse CD28	BioXcell	BE0015	2-5 µg/ml / T cell activation
Anti-human CD3 (OKT-3)	BioXcell	BE0001	1 µg/ml / PBMCs activation
Anti-human CD28	BioXcell	BE0291	1 µg/ml / PBMCs activation
Asparagine synthetase (G-10)	Santa Cruz	sc-365809	1:250 / IB
anti-actin	Santa Cruz	sc47778	1:1000 / IB

ATF-4 (D4B8) Rabbit mAb #	Cell Signaling	11815S	1:1000 / IB
NRF2 (D1Z9C) XP	Cell Signaling	12721S	1: 500 / Confocal
NRF2 (A 10)	Santa Cruz	365949	1:1000 / IB
DAPI	Vector Laboratories	EW-93952-27	20 ul/Slide
Anti-mouse IgG, HRP-linked Antibody	Cell Signaling	7076	1:3000 / IB
Anti-rabbit IgG, HRP-linked Antibody	Cell Signaling	7074	1:3000 / IB
Anti-Mouse IgG (H+L), (Alexa Fluor® 647)	Cell Signaling	4410S	1:1000 / Confocal
Recombinant Murine IL-12 p70	Peprotech	210-12	5 ng/ml / T cell polarization
Recombinant Murine IL-2	Peprotech	212-12 H1111	5 ng/ml / T cell activation/polarization

Supplementary table S2. List of cell culture reagents and chemicals

**Cell culture reagents and chemicals**			
**Name of the chemical/reagent**	**Vendor**	**Catalog #**	**Concentration**
H-2D(b) human gp100 25-33 peptide	Genescript	RP20344	1μM
RPMI 1640 Medium Modified w/o L-Glutamine, w/o Amino acids, Glucose (Powder)	US Biologicals	R9010-01	NA
RPMI 1640 Medium	Corning	10-040-CVR	NA
RPMI medium modified w/o asparagine, glutamine, glucose	Gibco	ME18399P1	NA
2-Mercaptoethanol	Gibco	M6250	0.05-0.15mM
Cell Stimulation Cocktail (plus protein transport inhibitors) (500X)	eBioscience	00-4975-93	1:500
Lipofectamine 2000 Transfection Reagent	Invitrogen	11668019	8 μl/ml
7-amino-actinomycin D(7AAD)	Biolegend	420404	5 μl/sample
D-(+)-Glucose	Sigma-Aldrich	G7021	11mM
L-Glutamine ($^{13}C_5$, 99%)	Cambridge Isotope Laboratories, Inc.	CLM-1822-H-PK	4mM
MC 228 D-Glucose, [1-^{14}C]-	Moravek Biochemicals	742-018-0555-A20110726-SBA	1μCi
MT 911 2-Deoxy-D-glucose, [1,2-3H(N)]-	Moravek Biochemicals	257-078-025-A-20130528-TNG	1μCi
MC 1124 L-Glutamine, [^{14}C(U)]-	Moravek Biochemicals	945-012-281-A20170301-SBA	0.2 μCi
Glucose, D-[5-^{3}H(N)]	American Radiolabeled Chemicals	131002	1μCi
MC 1124 L-Glutamine, [^{14}C(U)]-	Moravek Biochemicals	945-012-281-A20170301-SBA	1 μCi
L-Asparaginase	prospec	ENZ-287	10 IU for invivo 0.01IU for invitro
L-Asparagine	Sigma	A4159	0.3mM
FCCP	Sigma	C2520	2μM
Poly-D-Lysine	Millipore	A3890401	A3890401
Slide-A-Lyzer Dialysis Cassettes	Thermo Scientific	87721	NA
Matrigel matrix	Corning	CB40234	NA
Isobutylhydroxylamine hydrochloride	TCI	I0387	20 mg/mL

Seahorse XF96 Cell Culture Microplates	Agilent	101085	NA
E-plates	Agilent	300600900	NA
**Critical commercial assays**	**Vendor**	**Catalog #**	
BrdU kit APC	BioLegend	370706	
MojoSort™ Mouse CD8 Naïve T Cell Isolation Kit	Biolegend	480044	
Protein Synthesis Assay Kit	Cayman	601100	
ELISA MAX™ Deluxe Set Mouse TNF-α-	Biolegend	430904	
ELISA MAX™ Deluxe Set Mouse IFN-γ	Biolegend	430804	
Mouse Granzyme B DuoSet ELISA	R & D	DY1865-05	
LEGENDplex™ human CD8/NK panel	Biolegend	740267	
Seahorse XF- real-time ATP rate assay kit	Agilent	103592	
Foxp3 / Transcription Factor Staining Buffer Set	e-Bioscience	00-5523-00	
L-Arginine Assay Kit	Bio vision	K749	
CellTrace™ Violet Cell	Invitrogen	C34557	
carboxyfluorescein diacetate succinimidyl ester (CFSE)	Invitrogen	65-0850-84	
Pierce™ BCA Protein Assay kit	Thermo fisher scientific	23227	
M-MLV Reverse Transcriptase	Invitrogen	28025013	
Rneasy Mini Kit	Qiagen	NC9677589	
Quick-RNATM MiniPrep Kit	Zymo Research	R1055	
MitoTracker™ Green FM	Invitrogen	M7514	
Seahorse XF Media, Supplements & Calibrant	Agilent	103681	
CellTiter-Glo	Promega	G9241	
**Biological samples**			
Buffy coats from healthy human subjects	Central Ohio Region American Red Cross	NA	
**Experimental models: Organisms/strains**			
WT mouse: C57BL/6NJ,	Jackson Laboratory	Strain #: 000664	
Pmel-1 mouse:B6.Cg-Thy1a/CyTg(TcraTcrb)8Rest/J	Jackson Laboratory	Strain #:005023	
NRF2 KO mouse: B6.129X1-Nfe2l2tm1Ywk/J	Jackson Laboratory	Strain #:017009	
Rag1-/- mouse: B6.129S7-Rag1tm1Mom/J	Jackson Laboratory	Strain #:002216	
WT mouse: B6.PL-Thy1a/CyJ	Jackson Laboratory	Strain #: 000406	
ATF4 KI mouse: B6;129X1-Gt(ROSA)26Sortm2(ATF4)Myz/J	Jackson Laboratory	Strain #:029394	
B-NDG mouse: NOD.CB17-Prkdcscid IL2rgtm1/BcgenHsd	Envigo	NA	
ATF4 KO mouse: ATF4fl/fl	Dr. Christopher Adams (Mayo Clinic)	(*21*)	
ASNS KO mouse: C57BL/6N-Asns tm1a(EUCOMM)Wtsi/H) mice	MRC Harwell Institute	Stock code: IVF/5248	
**Experimental models: Cell lines**			
B16F10	American Type Culture Collection	CRL-6475	
LAN-1	Dr. Xiaotong Song (Baylor College of Medicine)		
B16-gp100	Dr. Nicholas Restifo (NIH)	(*49*)	

CMT167	Dr. Williams Terence (OSU)	
**Recombinant DNA**		
pCLIP-ALL-EFS-ZsGreen (TELA1013-Negative Control)	Transomic	CAMS 1013
pCLIP-ALL-EFS-ZsGreen (TEVM-1207871-pCLIP-ALL-EFS-ZsGreen)	Transomic	CAMS 1013
pCLIP-ALL-EFS-ZsGreen (TEVM-1140729-pCLIP-ALL-EFS-ZsGreen)	Transomic	CAMS 1013
pCLIP-ALL-EFS-ZsGreen (TEVM-1073587-pCLIP-ALL-EFS-ZsGreen)	Transomic	CAMS 1013
pMIG-Nrf2ΔNeh2	This paper	

Supplementary table S3. List of primers

qRT-PCR Primers	**Forward Primer Sequence (5' → 3')**	**Reverse Primer Sequence (5' → 3')**
IFN-γ	ATGAACGCTACACACTGCATC	CCATCCTTTTGCCAGTTCCTC
TNF-α	GCCCAGGCAGTCAGATCATCT	TTGAGGGTTTGCTACAACATGG
Granzyme B	GACCCAGCAGTTTATCCCTGT	CTGGGCCTTGTTGCTAGGTA
ATF4	ATGACCGAAATGAGCTTCCTG	GCTGGAGAACCCATGAGGT
ASNS	GGAAGACAGCCCCGATTTACT	AGCACGAACTGTTGTAATGTCA
Nrf2	TCAGCGACGGAAAGAGTATGA	CCACTGGTTTCTGACTGGATGT
Tubulin	TTCTGGTGCTTGTCTCACTGA	CAGTATGTTCGGCTTCCCATTC
Perforin	AGCACAAGTTCGTGCCAGG	GCGTCTCTCATTAGGGAGTTTTT
IL15	ACATCCATCTCGTGCTACTTGT	GCCTCTGTTTTAGGGAGACCT
IL6	TAGTCCTTCCTACCCCAATTTCC	TTGGTCCTTAGCCACTCCTTC
IL2	GTGCTCCTTGTCAACAGCG	GGGGAGTTTCAGGTTCCTGTA
IL23	ATGCTGGATTGCAGAGCAGTA	ACGGGGCACATTATTTTTAGTCT
IL4	GGTCTCAACCCCCAGCTAGT	GCCGATGATCTCTCTCAAGTGAT
IL13	CCTGGCTCTTGCTTGCCTT	GGTCTTGTGTGATGTTGCTCA
IL9	ATGTTGGTGACATACATCCTTGC	TGACGGTGGATCATCCTTCAG
IL22	ATGAGTTTTTCCCTTATGGGGAC	GCTGGAAGTTGGACACCTCAA
IL12	ATGGCTGCTGCGTTGAGAA	AGCACTCATAGTCTGTCTTGGA
T-bet	AGCAAGGACGGCGAATGTT	GTGGACATATAAGCGGTTCCC
TCF	CGAAAAGTTCCTCCGGGTTTG	CGTAGCCGGGCTGATTCAT
PRDM	TTCTCTTGGAAAAACGTGTGGG	GGAGCCGGAGCTAGACTTG
mouse COI	GCCCCAGATATAGCATTCCC	GTTCATCCTGTTCCTGCTCC
G6PDx	CACAGTGGACGACATCCGAAA	AGCTACATAGGAATTACGGGCAA
G6PD2	AGGTGACCCTAAGCCGGAC	AGGTTTCTTTGGGTAGAAGACCA
PGD	ATGGCCCAAGCTGACATTG	GCACAGACCACAAATCCATGAT
TKT	ATGGAAGGTTACCATAAGCCAGA	TGCAGCATGATGTGGGGTG
CAD	CTGCCCGGATTGATTGATGTC	GGTATTAGGCATAGCACAAACCA
DHODH	TCTTCACCTCTTACCTGACAGC	CATGTTGGAGTCCTGAAACGTA
UMPS	GTCACCGAGCTGTATGACGTG	GGTAACGCTGTATAAGGAACTCC
CPS1	CTTCCCACGCTGGAGCAGCC	CCGAAGCACGCCACCTCTCC
PPAT	GCGAGGAATGTGGTGTGTTTG	TTTAGGCACTGCACTCCCATC
PNP	ATCTGTGGTTCCGGCTTAGGA	TGGGGAAAGTTGGGTATCTCAT
IMPDH1	GGCTACGTTCCCGAGGATG	GGCTGATGTCAGGTCCACTT
PRPS	ATGCCTAACATCGTGCTCTTC	GATCTCGACACTGGTCTCCTG
PFAS	TCTCCTTCATGGGTCTCCCC	AGGCCCTGTTTCTCCTCTATC
ADSL	AGCCGCGAGATGTGTTTCTT	TCAATGTTGTTCAGGTTCGACTT
ADSS	ACACGGGGTAGAGAATTTGGA	GGTAAGGGCCAACGCAGTA

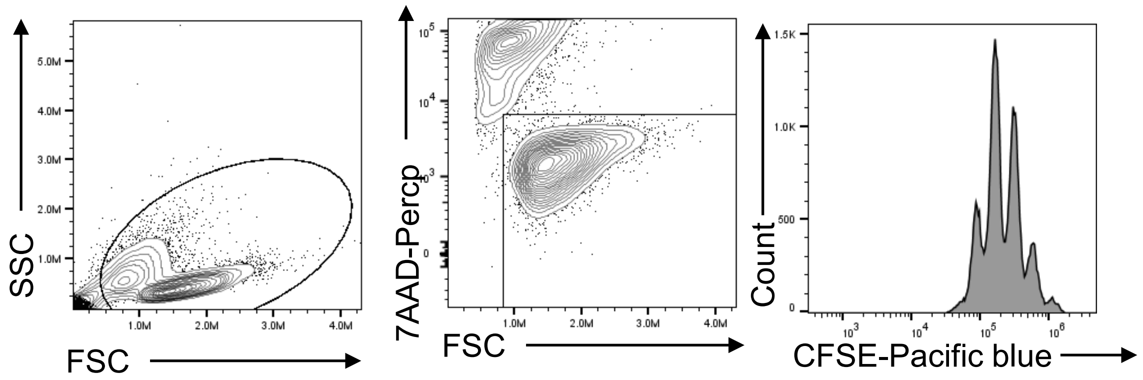

Supplemental Figure 1. Gating Strategy for cell proliferation. FSC-SSC-H gating was used as preliminary gating for the lymphocyte population, followed by live cell gating and CFSE.

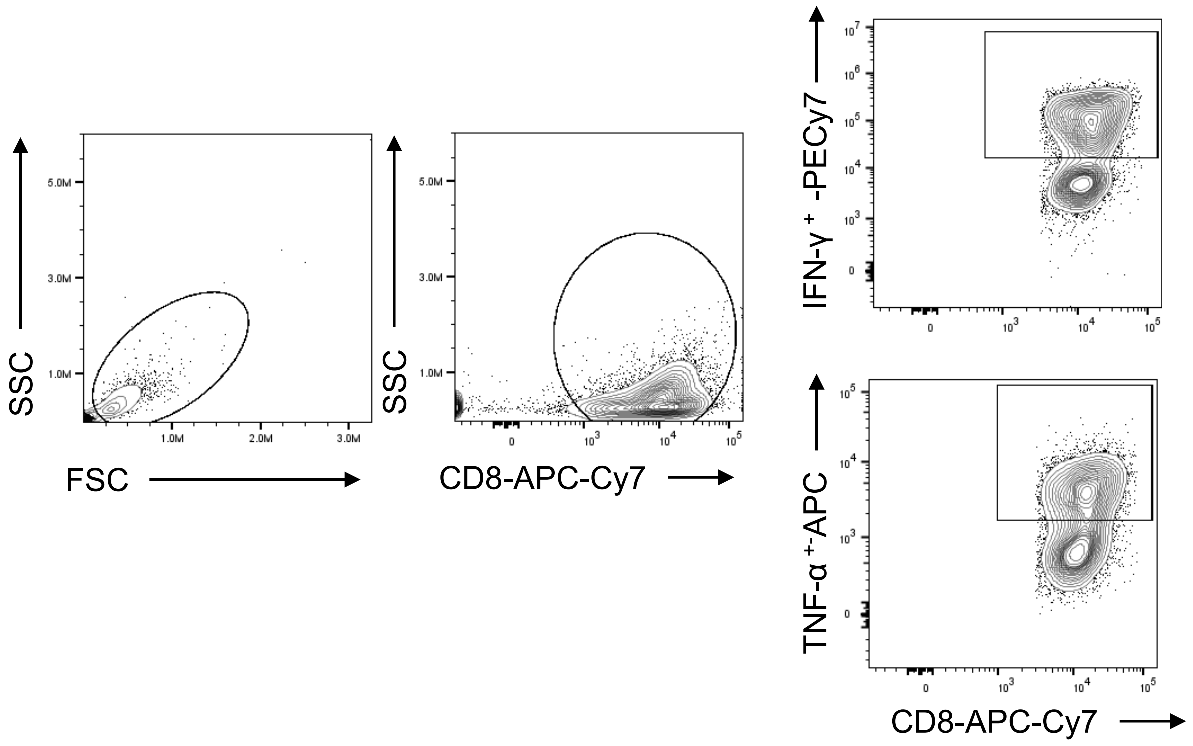

Supplemental Figure 2. Gating Strategy for analysis of cytokine production. FSC-SSC-H gating was used as preliminary gating for the lymphocyte population, followed by CD8+ staining and intracellular production of IFN-γ and TNF-α.
