# Supplementary material for: Asparagine restriction enhances CD8+ T cell metabolic fitness and antitumoral functionality through an NRF2-dependent stress response
Source: Nat Metab. 2023 Aug 7;5(8):1423–39. doi: 10.1038/s42255-023-00856-1 (PMC10447245; doi:10.1038/s42255-023-00856-1)
Supplement: Supplementary file 2 — Reporting Summary [file 42255_2023_856_MOESM2_ESM.pdf]

nature portfolio

Corresponding author(s): Ruoning Wang

Last updated by author(s): Jun 19, 2023

Reporting Summary

Nature Portfolio wishes to improve the reproducibility of the work that we publish. This form provides structure for consistency and transparency in reporting. For further information on Nature Portfolio policies, see our Editorial Policies and the Editorial Policy Checklist.

Statistics

For all statistical analyses, confirm that the following items are present in the figure legend, table legend, main text, or Methods section.

n/a	Confirmed	
☐	☒	The exact sample size (*n*) for each experimental group/condition, given as a discrete number and unit of measurement
☐	☒	A statement on whether measurements were taken from distinct samples or whether the same sample was measured repeatedly
☐	☒	The statistical test(s) used AND whether they are one- or two-sided<br>*Only common tests should be described solely by name; describe more complex techniques in the Methods section.*
☒	☐	A description of all covariates tested
☒	☐	A description of any assumptions or corrections, such as tests of normality and adjustment for multiple comparisons
☐	☒	A full description of the statistical parameters including central tendency (e.g. means) or other basic estimates (e.g. regression coefficient) AND variation (e.g. standard deviation) or associated estimates of uncertainty (e.g. confidence intervals)
☐	☒	For null hypothesis testing, the test statistic (e.g. *F*, *t*, *r*) with confidence intervals, effect sizes, degrees of freedom and *P* value noted<br>*Give P values as exact values whenever suitable.*
☒	☐	For Bayesian analysis, information on the choice of priors and Markov chain Monte Carlo settings
☒	☐	For hierarchical and complex designs, identification of the appropriate level for tests and full reporting of outcomes
☒	☐	Estimates of effect sizes (e.g. Cohen's *d*, Pearson's *r*), indicating how they were calculated

Our web collection on statistics for biologists contains articles on many of the points above.

Software and code

Policy information about availability of computer code

Data collection	Flow cytometry by using Novocyte (ACEA Biosciences) software (version 2000); quantitative PCR by using BIO-RAD CFX284TM Real-Time PCR Detection System; RNA-seq quality control and adapter trimming were accomplished using the FastQC (version 0.11.3) and Trim Galore (version 0.4.0) software packages, trimmed reads were mapped to the Genome Reference Consortium GRCm38 (mm10) murine genome assembly using TopHat2 (version 2.1.0), and feature counts were generated using HTSeq (version 0.6.1); 13C-tracer data by using Gas Chromatography-Mass Spectrometry (GC-MS) as standard method; medium metabolites by using Liquid Chromatography-Mass Spectrometry (LC-MS) (metabolon), NMR, or bioanalyzer (YSI, version 2900) as standard method; Oxygen consumption rate (OCR) by using seahorse XFe96 Analyzer (Agilent Technologies, version 2.6.1) and tumor killing was assessed using eSight (Agilent Technologies, version 1.0.3).
Data analysis	Flow cytomertric analysis were performed with FlowJo software (TreeStar, version 10.8.1); RNAseq analysis were performed using the DESeq2 package (version 1.16.1) in R, with the default Benjamini-Hochberg p-value adjustment method, the Ingenuity Pathway Analysis (IPA) software (QIAGEN, version 01-20-04), Metaboanalyst (version 5.0), the Gene Set Enrichment Analysis (GSEA) software (UC San Diego, BROAD Ins. version 4.1.0), ImageJ (version 1.53T)Oxygen consumption rate (OCR) were analysis by using the Seahorse Wave Software (Seahorse, Agilent Technologies. version 2.6); percentage cytolysis were calculated by RTCA PRO (Agilent Technologies. version 2.6.0); Statistical data analysis and generation of graphs by using GraphPad Prism (version 9).

For manuscripts utilizing custom algorithms or software that are central to the research but not yet described in published literature, software must be made available to editors and reviewers. We strongly encourage code deposition in a community repository (e.g. GitHub). See the Nature Portfolio guidelines for submitting code & software for further information.

Data

Policy information about availability of data

All manuscripts must include a data availability statement. This statement should provide the following information, where applicable:

- Accession codes, unique identifiers, or web links for publicly available datasets
- A description of any restrictions on data availability
- For clinical datasets or third party data, please ensure that the statement adheres to our policy

Raw RNA-seq datasets generated for this study can be found in the GEO accession GSE201870 (https://www.ncbi.nlm.nih.gov/geo/subs/). The authors declare that all other data (including the Metabolon and LC-MS metabolomics data) and materials supporting the findings of this study are available within the article (and supplementary/ extended information files).

Field-specific reporting

Please select the one below that is the best fit for your research. If you are not sure, read the appropriate sections before making your selection.

☒ Life sciences ☐ Behavioural & social sciences ☐ Ecological, evolutionary & environmental sciences

For a reference copy of the document with all sections, see nature.com/documents/nr-reporting-summary-flat.pdf

Life sciences study design

All studies must disclose on these points even when the disclosure is negative.

Sample size	Sample sizes determined on the basis of previous experience in previous experiments. For Metabolon, LC-MS, GS-MS and RNA seq studies 3 independent samples for determination (referring Ratnikov, B. et al. Bioinfornatics 2006; Bunk, B. et al. Bioinfornatics 2006; Evans, A.M. et al. Anal Chem 2009); for in vitro experiments , 3-6 independent samples were used and for in vivo adoptive transferred experiment, 3-5 and tumor xenograft, 5-10 independent mice were used (referring Wang,T et al. Nat Metab 2020; Wu, R. et al. Sci Adv 2020; Chen, X. et al. Sci Imm 2022).
Data exclusions	No excluded from the analysis.
Replication	All experiments were conducted with at least two independent experiments and multiple biological replicates (except metabolomics was performed one time and 3 biological replicates), and the details was provided in corresponding figure legends.
Randomization	All studies were performed on age and gender matched animals. Litter-mate animals were randomized prior to experiments. Samples for in vitro experiments were not randomized because they were defined groups by genotype and treatments.
Blinding	Investigators were not blinded during the data collection and data analysis because all analysis were performed using same gating as control under the same condition.

Reporting for specific materials, systems and methods

We require information from authors about some types of materials, experimental systems and methods used in many studies. Here, indicate whether each material, system or method listed is relevant to your study. If you are not sure if a list item applies to your research, read the appropriate section before selecting a response.

Materials & experimental systems

n/a	Involved in the study	
☐	☒	Antibodies
☐	☒	Eukaryotic cell lines
☒	☐	Palaeontology and archaeology
☐	☒	Animals and other organisms
☒	☐	Human research participants
☒	☐	Clinical data
☒	☐	Dual use research of concern

Methods

n/a	Involved in the study	
☒	☐	ChIP-seq
☐	☒	Flow cytometry
☒	☐	MRI-based neuroimaging

Antibodies

Antibodies used	Flowcytomtery antibodies<br>APC anti-mouse IFN-γ Antibody BioLegend 505810, Cat # 1:200 dilution<br>PE/Cyanine7 anti-mouse IFN-γ Antibody BioLegend 505826,Cat # 1:200 dilution<br>APC Anti-mouse TNF-α BioLegend 506308,Cat # 1:200 dilution

APC/Cyanine7 anti-mouse CD8a Antibody BioLegend 100714,Cat # 1:300 dilution
APC anti-mouse TCR β chain Antibody BioLegend 109211,Cat # 1:100 dilution
PE NRF2 rabbit monoclonal Antibody Cell signaling 14409,Cat # 1:100 dilution
APC/Cyanine7 anti-mouse thy1.1 Antibody BioLegend 202520,Cat # 1:100 dilution
Percp anti-mouse thy1.2 Antibody BioLegend 140316,Cat # 1:100 dilution
CD25 Monoclonal Antibody (PC61.5), PE eBioscience 12-0251,Cat # 1:300 dilution
FITC anti-mouse CD8 Antibody BioLegend 100705,Cat # 1:200 dilution
APC/Cyanine7 anti-human CD8a Antibody BioLegend 300926,Cat # 1:200 dilution
PE/Cyanine7 anti-human IFN-γ Antibody BioLegend 506518,Cat # 1:200 dilution
PE anti-human TNF-α Antibody BioLegend 502909,Cat # 1:200 dilution
PE/Cyanine7 anti-mouse CD4 BioLegend 100422,Cat # 1:200 dilution
PE Glut1 Antibody Novus Biologicals NB110-39113, Cat # 1:100 dilution
PE Asparagine synthetase Antibody Santa Cruz SC-365809 1:100 dilution
T cell activation and immunothyerapy antibodies:
InVivoMAb anti m PD-L1 BioXcell BE0101,Cat # 200 μg per dose
InVivoMAb anti m PD-1 BioXcell BE0146,Cat # 200 μg per dose
InVivoMAb Rat IgG2b Isotype control BioXcell BE0090,Cat # 200 μg per dose
InVivoMAb anti-mouse CD3 BioXcell BE0001,Cat # 2-5 μg/ml
InVivoMAb anti-mouse CD28 BioXcell BE0015,Cat # 2-5 μg/ml
Anti-human CD3 (OKT-3) BioXcell BE0001,Cat # 1 μg/ml
Anti-human CD28 BioXcell BE0291,Cat # 1 μg/ml
Recombinant Murine IL-12 p70 Peprotech 210-12,Cat # 5 ng/ml
Recombinant Murine IL-2 Peprotech 212-12 H1111,Cat # 5 ng/ml
Confocal/IB antibodies:
Asparagine synthetase (G-10) Santa Cruz sc-365809,Cat # 0.215277778
anti-actin Santa Cruz sc47778,Cat # 0.736111111
ATF-4 (D4B8) Rabbit mAb # Cell Signaling 11815S,Cat # 0.736111111
NRF2 (D1Z9C) XP Cell Signaling 12721S,Cat # 0.388888889
NRF2 (A 10) Santa Cruz 365949,Cat # 0.736111111
Anti-mouse IgG, HRP-linked Antibody Cell Signaling 7076,Cat # 2.125
Anti-rabbit IgG, HRP-linked Antibody Cell Signaling 7074,Cat # 2.125
Anti-Mouse IgG (H+L), (Alexa Fluor® 647) Cell Signaling 4410S,Cat # 0.736111111

Validation

Reactivity of above antibodies are commercially available and validated for indicated applications, all information on manufacturer's homepage and have been extensively referenced in literatures.
https://bxcell.com/
https://www.biolegend.com/
https://www.scbt.com/home
https://www.thermofisher.com/us/en/home/life-science/antibodies/ebioscience
https://www.cellsignal.com/
https://www.sigmaaldrich.com/US/en

Eukaryotic cell lines

Policy information about cell lines

Cell line source(s)

B16F10 from ATCC, LAN-1 from Dr. Xiaotong Song (Wang,T et al. Nat Metab 2020;), B16-gp100 from Dr. Nicholas Restifo (Vodnala,S.K et al. Science 2019) and CMT167 from Dr. Williams Terence (Ismail et al. Cancer Res 2000).

Authentication

Cell lines used in this study were purchased from ATCC or from other research groups and were not authenticated.

Mycoplasma contamination

Cell lines used in this study were not tested for Mycoplasma contamination.

Commonly misidentified lines
(See ICLAC register)

No commonly misidentified cell line was used in this study.

Animals and other organisms

Policy information about studies involving animals; ARRIVE guidelines recommended for reporting animal research

Laboratory animals

WT mouse: C57BL/6NJ, WT mouse: B6.129S4-Ifnγtm3.1Lky/J, Pmel-1 mouse:B6.Cg-Thy1a/CyTg(TcraTcrb)8Rest/J, NRF2 KO mouse: B6.129X1-Nfe2l2tm1Ywk/J,Rag1-/- mouse: B6.129S7-Rag1tm1Mom/J,CD45.1 mouse: B6.SJL-Ptprca Pepcb/BoyJ,ATF4 KI mouse: B6;129X1-Gt(ROSA)26Sortm2(ATF4)Myz/J, B-NDG mouse: NOD.CB17-Prkdcscid IL2rgtm1/BcgenHsd, ATF4 KO mouse: ATF4fl/fl ASNS KO mouse: C57BL/6N-Asns tm1a(EUCOMM)Wtsi/H) mice. Both male and female mice, with age-matched (6-12 weeks old) were used in the experiments. Mice were housed under controlled conditions: rodent housing rooms are kept at 73 degree Fahrenheit, with alarms set at 69 and 78 degrees, 30–70% relative humidity, and 12:12 light-dark cycle. Food and water was available for all animals. Low Fat diet were provided (Envigo 2920, the irradiated form of 2020X*). Mice were maintained and euthanized (by carbon dioxide asphyxiation followed by cervical dislocation) under protocols approved by the Institutional Animal Care and Use Committee of the Research Institute at Nationwide Children's Hospital (IACUC; protocol number AR13-00055). *https://insights.envigo.com/hubfs/resources/data-sheets/2020x-datasheet-0915.pdf

Wild animals

This study does not include any wild animals.

Field-collected samples	This study does not include samples collected from the field.
Ethics oversight	Animal protocols were approved by the Institutional Animal Care and Use Committee of the Research Institute at Nationwide Children's Hospital.

Note that full information on the approval of the study protocol must also be provided in the manuscript.

Flow Cytometry

Plots

Confirm that:

☒ The axis labels state the marker and fluorochrome used (e.g. CD4-FITC).

☒ The axis scales are clearly visible. Include numbers along axes only for bottom left plot of group (a 'group' is an analysis of identical markers).

☒ All plots are contour plots with outliers or pseudocolor plots.

☒ A numerical value for number of cells or percentage (with statistics) is provided.

Methodology

Sample preparation	For T cell isolation from mouse, spleen and lymph nodes were passed through 70 micron filters; Mononuclear cells from peripheral blood (PBMCs) were isolated from buffy coats by Ficoll/Hypaque density gradient centrifugation.
Instrument	Novocyte was used for collection of FACS data.
Software	FlowJo version 10.6
Cell population abundance	naive CD8 T cells was performed using kits (MojoSort, BioLegend) and no cell sorting were performed at this time.
Gating strategy	FSC-SSC-H gating was used as preliminary gating for lymphocyte population followed by analysis of effector T cells (CD8+) in all the experiments. Each population was gated based on the surface or intracellular markers as described in the manuscript.

☒ Tick this box to confirm that a figure exemplifying the gating strategy is provided in the Supplementary Information.
