# Supplementary material for: Asparagine restriction enhances CD8+ T cell metabolic fitness and antitumoral functionality through an NRF2-dependent stress response
Source: Nat Metab. 2023 Aug 7;5(8):1423–39. doi: 10.1038/s42255-023-00856-1 (PMC10447245; doi:10.1038/s42255-023-00856-1)

Confocal image for control- NRF2 (red)

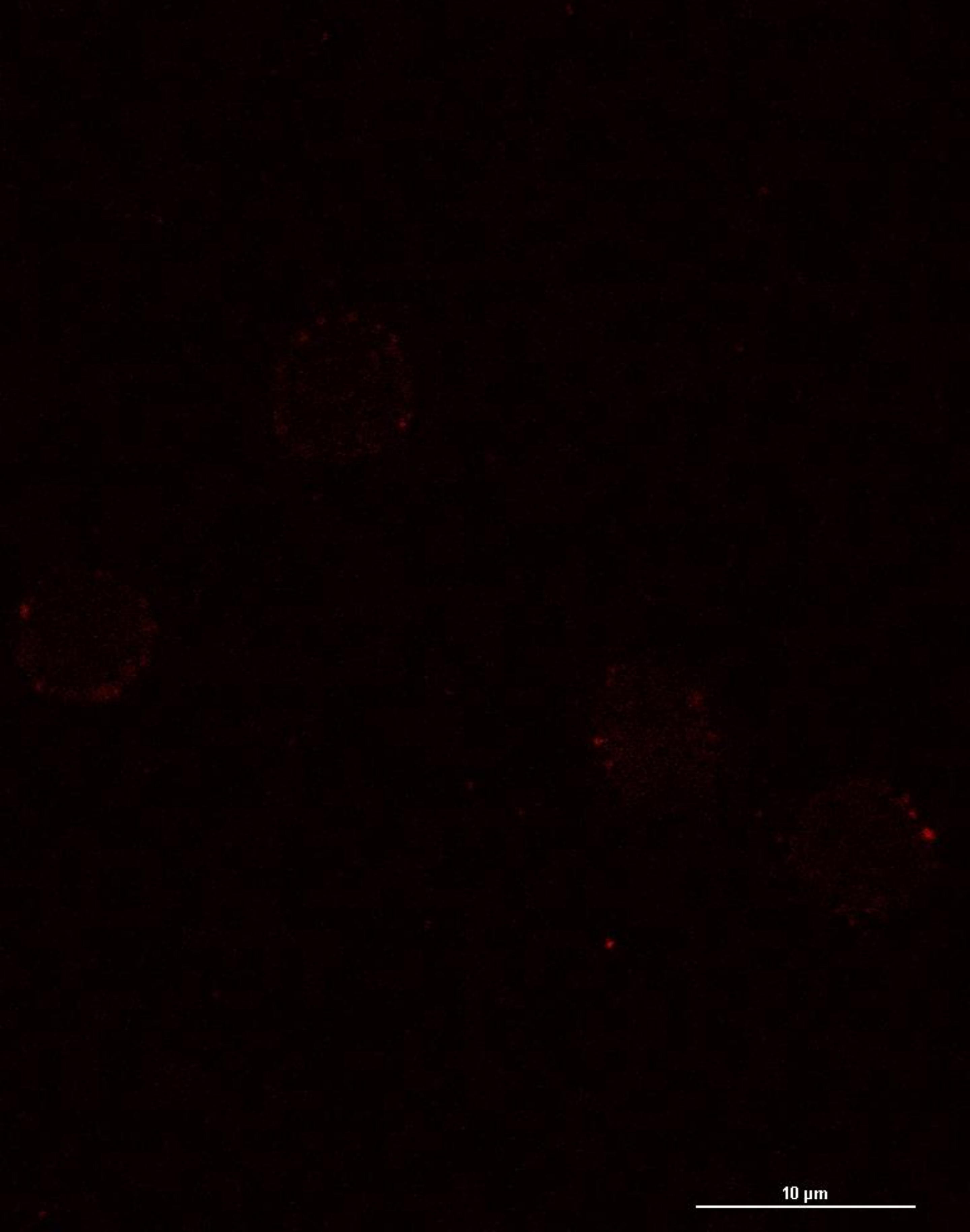

Confocal image for control- DAPI (blue)

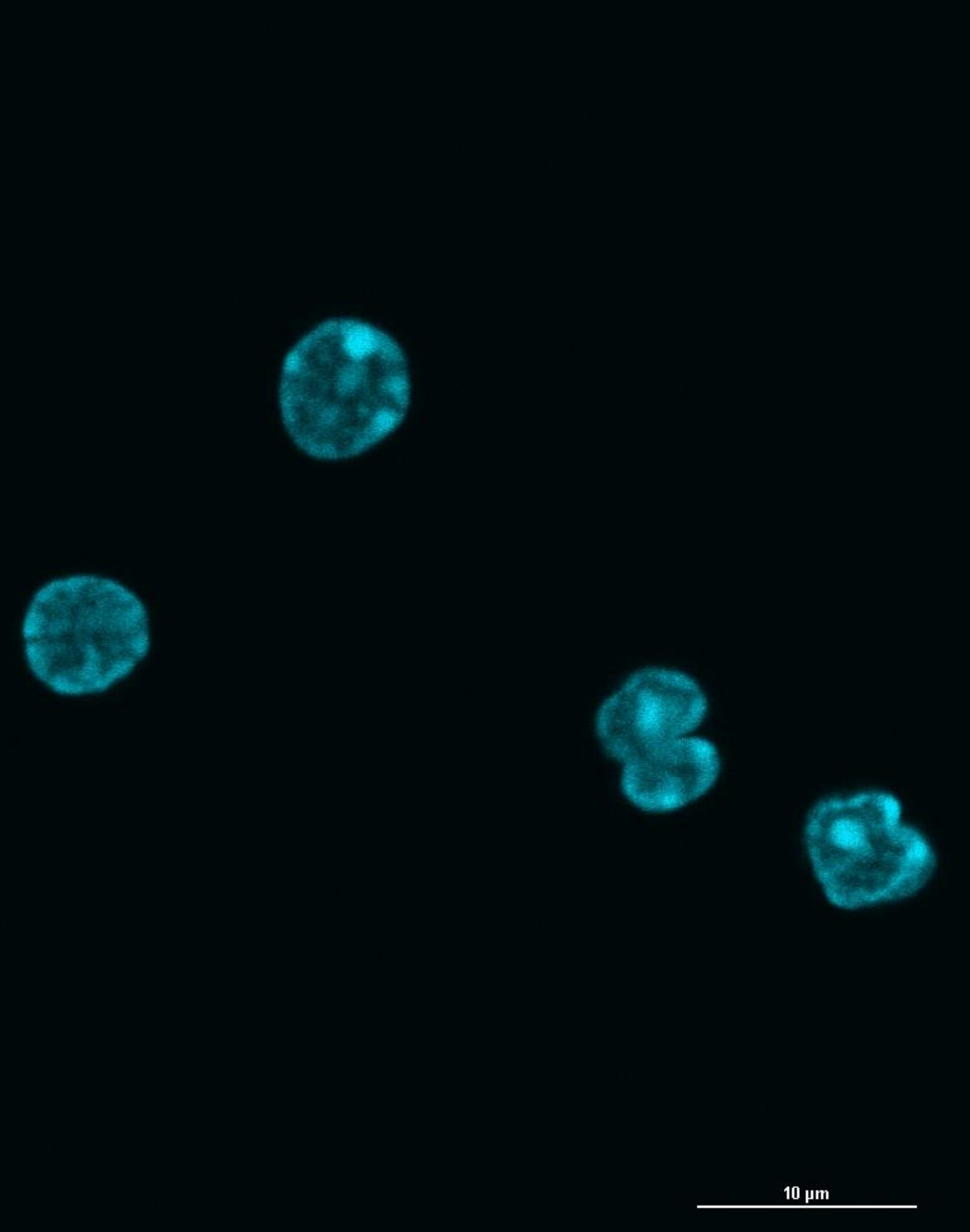

Confocal image for control- NRF2 (red)+DAPI (blue) merged

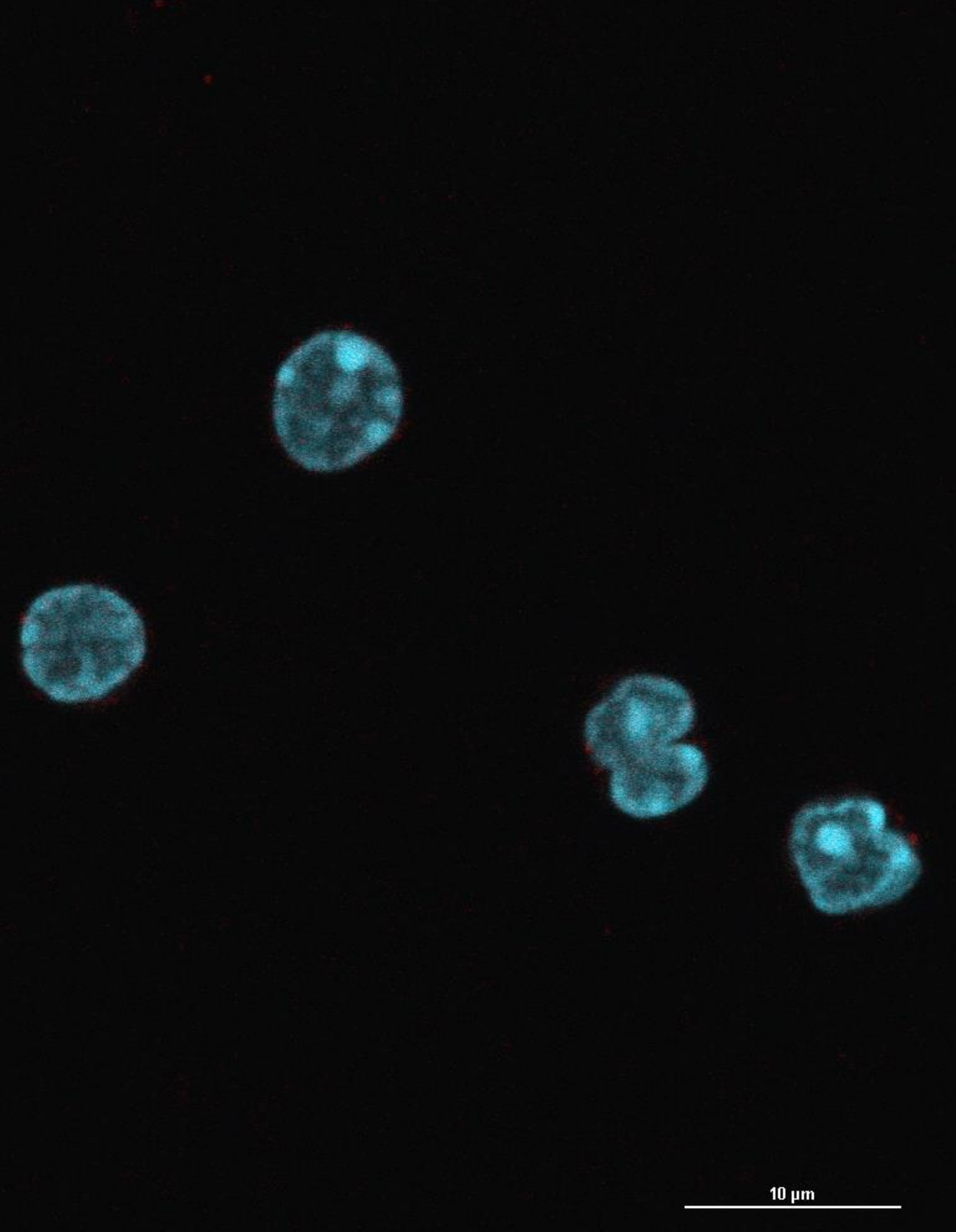

Confocal image for Asn restriction -NRF2 (red)

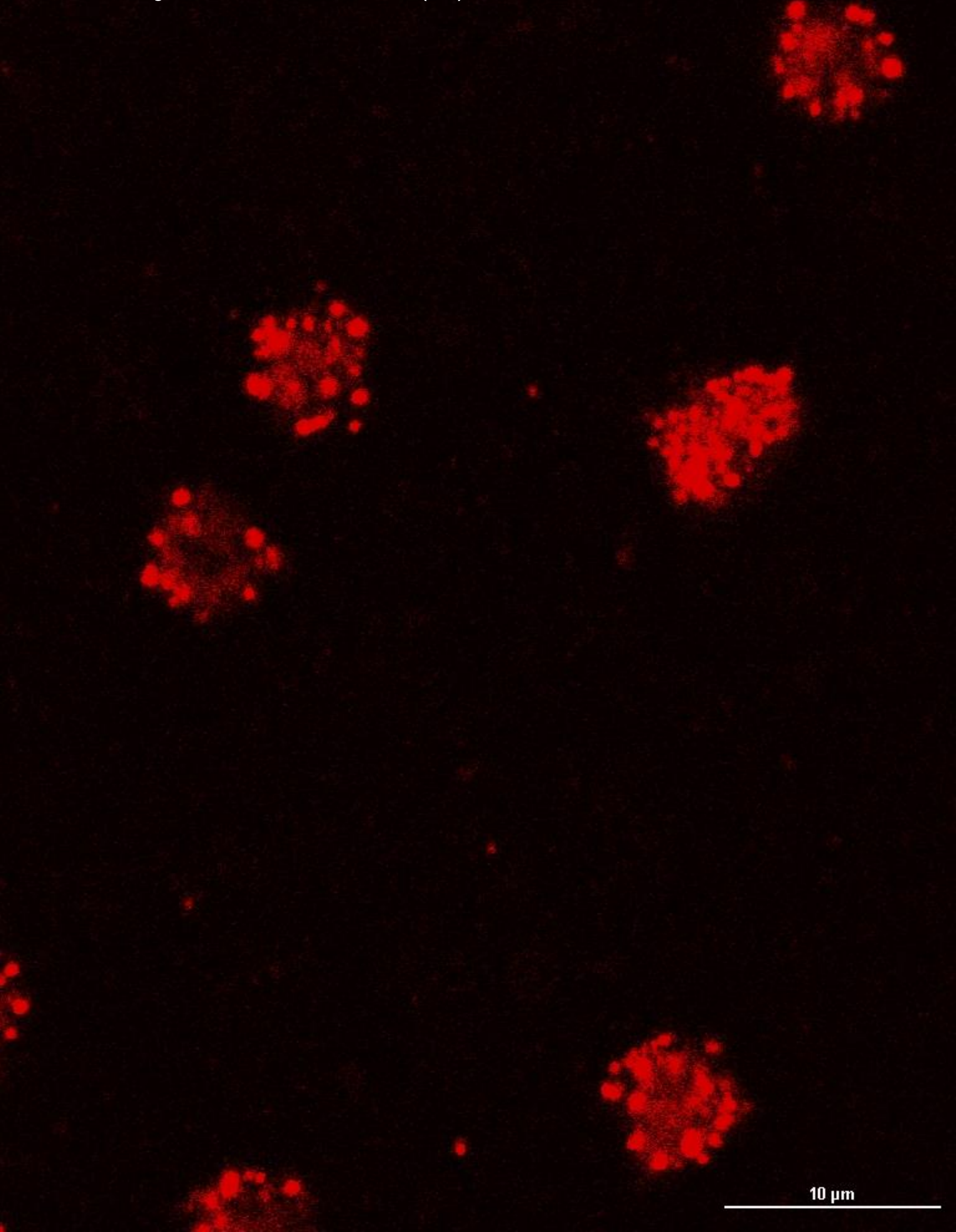

Confocal image for Asn restriction -DAPI (blue)

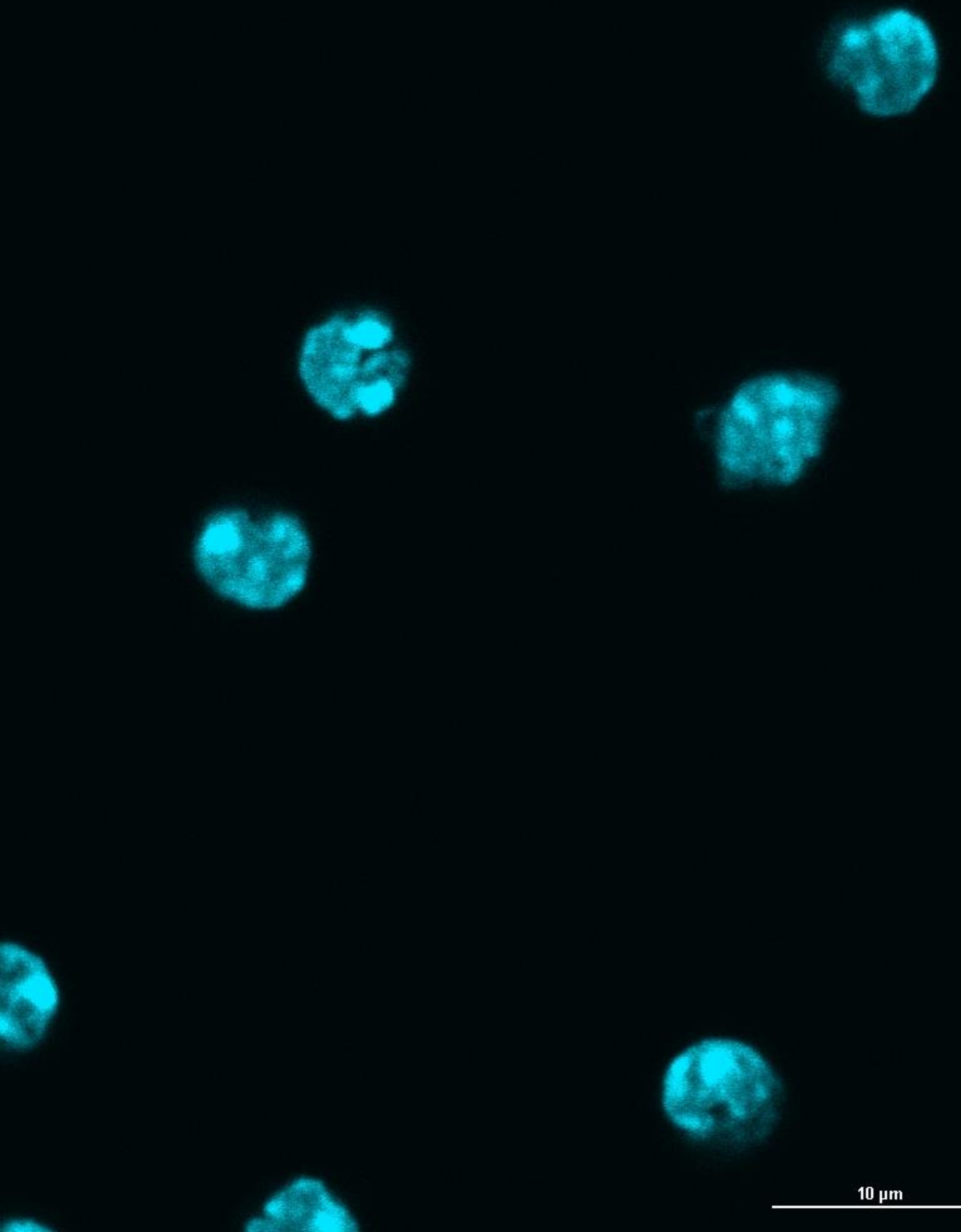

Confocal image for Asn restriction -NRF2 (red)+DAPI (blue) merged

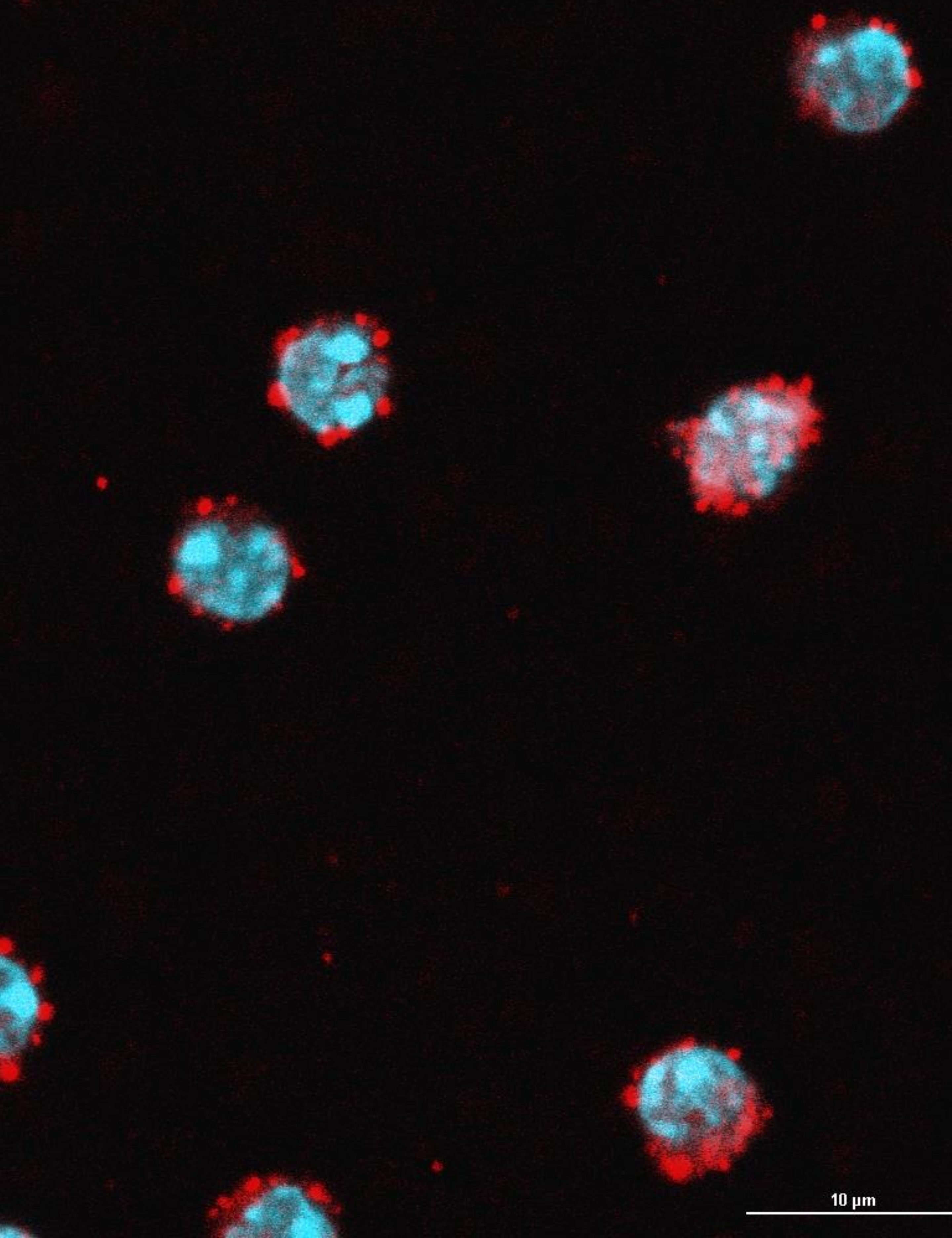

Supplement: Source Data Extended Data Fig. 4a — Unprocessed confocal images. [file 42255_2023_856_MOESM14_ESM.pdf]
